# Using machine learning to mine mental health diagnostic groups from emergency department presentations before and during the COVID-19 pandemic

**DOI:** 10.1007/s44192-023-00047-0

**Published:** 2023-11-06

**Authors:** Carly Hudson, Grace Branjerdporn, Ian Hughes, James Todd, Candice Bowman, Marcus Randall, Nicolas J. C. Stapelberg

**Affiliations:** 1 Bond University Faculty of Health Sciences and Medicine, Gold Coast, Queensland Australia; 2 Gold Coast Hospital and Health Service, Gold Coast, QLD Australia; 3 Centre for Data Analytics, Bond Business School, Bond University, Gold Coast, Queensland Australia

**Keywords:** Crisis care, Machine learning, Emergency department, Data mining, Health data analytics

## Abstract

**Purpose:**

The COVID-19 pandemic had a profound negative effect on mental health worldwide. The hospital emergency department plays a pivotal role in responding to mental health crises. Understanding data trends relating to hospital emergency department usage is beneficial for service planning, particularly around preparing for future pandemics. Machine learning has been used to mine large volumes of unstructured data to extract meaningful data in relation to mental health presentations. This study aims to analyse trends in five mental health-related presentations to an emergency department before and during, the COVID-19 pandemic.

**Methods:**

Data from 690,514 presentations to two Australian, public hospital emergency departments between April 2019 to February 2022 were assessed. A machine learning-based framework, *Mining Emergency Department Records, Evolutionary Algorithm Data Search* (MEDREADS), was used to identify suicidality, psychosis, mania, eating disorder, and substance use.

**Results:**

While the mental health-related presentations to the emergency department increased during the COVID-19 pandemic compared to pre-pandemic levels, the proportion of mental health presentations relative to the total emergency department presentations decreased. Several troughs in presentation frequency were identified across the pandemic period, which occurred consistently during the public health lockdown and restriction periods.

**Conclusion:**

This study implemented novel machine learning techniques to analyse mental health presentations to an emergency department during the COVID-19 pandemic. Results inform understanding of the use of emergency mental health services during the pandemic, and highlight opportunities to further investigate patterns in presentation.

**Supplementary Information:**

The online version contains supplementary material available at 10.1007/s44192-023-00047-0.

## Introduction

The COVID-19 pandemic has had a significant negative impact on mental health worldwide [1]. Lockdowns, public health restrictions, and associated disruptions to daily living increased stress, mental distress, and vulnerability in the population. Between 2020 and 2021, 3.5% of all presentations made to Australian emergency departments (EDs) related to mental health [2]. The ED remained open during COVID-19 despite public health restrictions and limited access to in-person primary health care services, providing a service for those who required emergency mental health care.

Data on mental health presentations is vital for health service planning as it can assist in retrospectively understanding trends and preparing for future pandemics [3]. In their analysis of mental health presentations to EDs in Western Australia from 2019 to 2020, Dragovic et al. [4] identified a decrease in cases at the onset of COVID-19 compared to 2019. Likewise, Jessup and Bramston [5] found a decrease in weekly presentations to the ED overall, not only for mental health, in Victoria as a result of the COVID-19 pandemic. Studies conducted internationally also found decreases in ED presentations for mental health crisis care during the COVID-19 pandemic, suggesting that these trends are not unique to Australia [6, 7].

Understanding variations in trends based among diagnostic groups is valuable as it informs which populations are more vulnerable and in need of more targeted interventions and specialised support. In Germany, Seifert et al. [8] found that during COVID-19, patients with affective disorders were less likely to present to an ED, whilst patients with personality and behavioural disorders were more likely to present to ED [8]. Additionally, both diagnostic groups were more likely to re-present to ED within a month of previous psychiatric care [8]. No differences in presentation rates for other diagnostic groups (e.g., substance use disorders, schizophrenia) were found [8].

A patient’s encounter with a health service generates substantial amounts of unstructured data, including triage data, clinical observations, and admission and discharge notes [9]. Large health service datasets provide insights into those accessing a health care service, as well as determining patterns of presentation [10]. Mental health presentations to public hospital emergency department settings also produces a large dataset, where significant shifts in mental health presentations have been observed since the onset of COVID-19 [11, 12]. Despite the opportunities that such large datasets present, there are several limitations. At present, there is no standardised method for data collection, input, or structuring across healthcare services within Australia, which can cause difficulty in extracting meaning and making comparisons across services [9]. Large datasets can also be costly to mine manually in terms of time and skilled human resources [13–17].

Machine learning (ML) refers to a wide range of techniques, which can automatically detect trends or patterns in a given dataset [18]. ML can be particularly useful when applied to problems which are otherwise too time or cost prohibitive to address. ML methodologies have been used to mine large volumes of unstructured data to extract and create structured data in relation to specific mental health presentation [19, 20]. Rozova et al. [21] used ML to detect self-harm presentations from 477,627 ED triage notes (2012–2018) at The Royal Melbourne Hospital, Australia. Data mining can automate the data extraction process, thereby reducing the cost of time and skill required to manually extract the data [10].

This research aimed to present a novel approach to classifying mental health presentations to EDs in Australia, using a suite of ML algorithms. The goal of these ML algorithms was to explore the trends in five mental health-related diagnostic groups in people presenting to EDs before and during, the COVID-19 pandemic in Australia:

1. *Suicidality*—presentations including suicide attempt, suicidal ideation, and non-suicidal self-injury.
2. *Eating disorder*—presentations relating to new or ongoing eating disorder diagnosis, such as anorexia nervosa, bulimia nervosa, binge eating disorder, and other specified eating disorders.
3. *Mania*—presentations including or relating to manic depression, bipolar disorder, or manic episodes.
4. *Psychosis*—presentations including or related to psychotic episodes or symptoms, and psychotic disorders such as schizophrenia or schizoaffective disorder.
5. *Substance Use*—presentations including accidental or intentional drug or alcohol intoxication, overdose, or other related problems.

## Methods

### Context and emergency department data analysed

This work was conducted under ethics exemption (EX/2022/QGC/85883). This study examined ED data within the Gold Coast Hospital and Health Service (GCHHS), a public health catchment within the metropolitan Gold Coast region, in Queensland, Australia, which provides specialised mental health services, in addition to general public health care to a population of approximately 640,000 people [22]. The Gold Coast is a transient population with a significant number of interstate and international visitors. This region has two public EDs, at Gold Coast University Hospital and Robina Hospital, which are both public hospitals providing tertiary level care. The former is recognised as the ED with the highest throughput in Australia [23]. All ED presentations between April 2019 to February 2022 were assessed (*N* = 690,514).

### Identification of mental health presentations with specific characteristics

An ML framework, called *Mining Emergency Department Records, Evolutionary Algorithm Data Search* (MEDREADS), was used to identify five mental health diagnostic groups in people presenting to the ED: (1) suicidality, (2) eating disorder, (3) mania, (4) psychosis, and (5) substance use. MEDREADS was developed using a range of variables extracted from Cerner FirstNet^®^, the ED patient records database used within GCHHS [24].

For each of the five diagnostic groups, an evolutionary algorithm was used to weight categorical variables as shown in Appendix A. Defined values for each of the included categorical variables have been provided in Appendix B. The presenting problem description, an unstructured text field consisting of the notes written by the triage intake nurse (e.g., *“BIB QPS causing disturbance outside high school. Mhx previous inpatient admission”*), was also included. These were chosen by a senior psychiatrist (NS) and selected if they were deemed to be of clinical significance.

The evolutionary based approach used in this study is a standard genetic algorithm which was tailored for a psychiatric classification task, to minimise deviation between observed and predicted values. A separate genetic algorithm implementation is developed for each of the five diagnostic groups. Each model assigns weights or scores to each of the variables in Appendix A. For a given presentation, the sum of the scores is compared to a model-specific threshold value to determine whether the presentation involves the diagnostic group or not. The threshold value was selected to achieve a specificity of 95% while maximising sensitivity.

The genetic algorithm was trained using 24,996 ED presentations obtained between 7th January 2020 and 31st August 2020 and validated on a dataset of 39,885 ED presentations obtained between 1st September 2020 and 28th November 2020. For the training dataset, each presentation was manually rated either 0 (no) or 1 (yes) for each of the diagnostic categories, by trained and supervised raters (CH and GB, supervised by NS). The presentations that were coded for were not mutually exclusive (i.e., one presentation could be coded for multiple diagnostic categories). The trained algorithm was then applied to the whole dataset.

### Statistical analysis

Statistical analyses were undertaken using Stata 17 and WinBUGS 1.4.3 [25, 26]. The MEDREADS diagnostic algorithm for each of suicidality, eating disorder, mania, psychosis, substance use was applied to a test data set consisting of 39,885 ED presentations for which a definitive diagnosis was available. The overall diagnostic test accuracy for each MEDREADS score was estimated by the area under the receiver operating characteristic (ROC) curve (AUC-ROC) and the optimal cut-point identified by Youden’s method. Sensitivity (*Se*) and specificity (*Sp*) were calculated with binomial exact 95% confidence intervals (CI). MEDREADS was then applied to each month of ED presentations from April 2019 to February 2022. Crude prevalence estimates for each month were calculated as the proportion (with logit 95% CI) of MEDREADS positive diagnoses. Unless a diagnostic test is perfect, false positives and false negatives will result in an inaccurate true prevalence estimate. If the true prevalence is small, the crude prevalence will likely be an overestimate.

To address this problem, we used a Bayesian approach with Gibbs sampling described by Joseph et al. [27] and Messam et al. [28] and undertaken in WinBUGS. We assumed that the number of positive test results (correct identification of mental health diagnostic groupings), *T*_*P*_, is binomially distributed. ${T}_{P}\sim B\left({P}_{C}, n\right)$, where *P*_*C*_ is the crude prevalence and $n$ is the total number in the population (ED presentations in a month). Crude prevalence is related to true prevalence, *P*_*T*_, *Se*, and *Sp* through the equation${P}_{C}={P}_{T}Se+\left(1-{P}_{T}\right)\left(1-Sp\right)$. For example, for a rare true prevalence such as 0.04 if *Se* and *Sp* are perfect, 1.0, the crude prevalence will be accurately estimated as 0.04. However, if *Se* and *Sp* are 0.95, still good for a diagnostic test, the crude prevalence estimate is 0.09, more than double the true prevalence. Prior estimates of *P*_*T*_, *Se*, and *Sp* are used in the analyses and were obtained from MEDREADS results from the test data set. These prior estimates are distributions around point estimates ranging from 0 to 1 and can be represented as beta distributions with hyperparameters *a* and *b*. Appropriate *a* and *b* hyperparameters were obtained using the beta parameters utility from Epitools [29] based on the mode (mean) and the 95th (*P*_*T*_ prior) or 5th (*Se* and *Sp* priors) percentile of the *P*_*T*_, *Se*, and *Sp* estimates from the test data set. For example, the *P*_*T*_ prior estimate for suicide prevalence was based on a mean of 0.029 and its 95th percentile, 0.031, from the test set data. Entering these values into the Epitools beta distribution utility, the hyperparameters *a* = 828.7 and *b* = 27,485.7 were obtained. Prior estimates of *P*_*T*_, *Se*, and *Sp* were entered into WinBUGS code along with their associated beta distribution hyperparameters, the observed number of positive diagnoses, and total ED presentations for each month for each diagnosis type. The posterior estimate of *P*_*T*_ (true prevalence estimate) was obtained as a median and 95% probability interval following the generation of 50,000 random samples. WinBUGS code for each diagnosis type is provided in the Supplementary file 1.

## Results

**Fig. 1:**
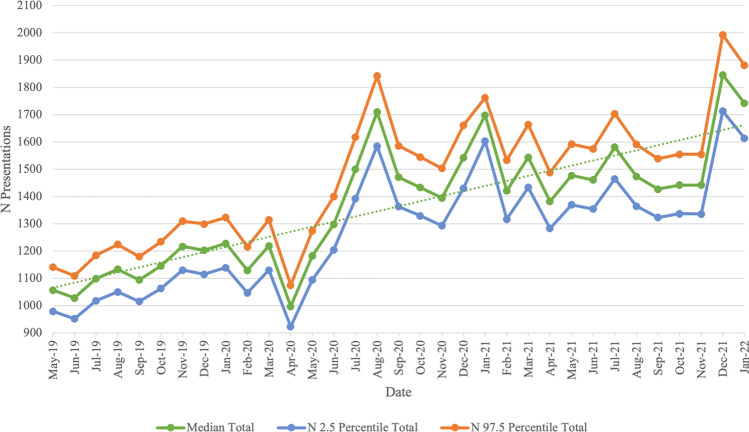
Bayesian Estimate (with 95% probability interval) of Presentations Made to Gold Coast EDs Across the Five Diagnostic Groups Between May 2019 and January 2022: Total Number Across All Diagnostic Groups (Note. Data was incomplete for April 2019 and February 2022, and is not displayed in the graph)

Between April 2019 and February 2022, a total of 46,849 presentations across the five diagnostic groups were identified. There was an increase in the overall number of presentations across the five diagnostic groups, from April 2019 (before the COVID-19 pandemic) (*N* = 599, 7.40%) to February 2022 (during the COVID-19 pandemic) (*N* = 1202, 7.00%) (refer to Fig. 1). The overall number of cases across the five diagnostic groups peaked in December 2021 (*N* = 1846, 5.51%). Total numbers of mental health presentations across the five diagnostic groups are provided Table 1. Table 2 gives sensitivity (*Se*) and specificity (*Sp*) for these estimates.

**Table 1:** Bayesian Estimate (with 95% probability interval) of Overall Presentations to the Gold Coast Emergency Departments, April 2019—February 2022

Month	2019		2020		2021		2022	
	Total ED presentations across all diagnostic groups*^	% Of all ED Presentations	Total ED presentations across all diagnostic groups*^	% Of all ED presentations	Total ED presentations across all diagnostic groups*^	% Of all ED presentations	Total ED presentations across all diagnostic groups*^	% Of all ED presentations
January	–	–	1228 (1139–1323)	7.70	1698 (1603–1763)	6.31	1742 (1614–1881)	5.15
February	–	–	1129 (1047–1216)	7.64	1421 (1317–1533)	6.84	1202 (1112–1295)	7.00
March	–	–	1219 (1130–1315)	7.02	1544 (1433–1664)	6.83	–	–
April	599 (555–647)	7.40	997 (923–1075)	7.17	1382 (1283–1488)	6.98	–	–
May	1057 (979–1141)	7.30	1182 (1094–1274)	7.01	1477 (1370–1592)	7.02	–	–
June	1028 (952–1109)	7.28	1298 (1205–1400)	6.75	1461 (1354–1575)	6.50	–	–
July	1099 (1018–1185)	7.25	1500 (1392–1618)	6.55	1581 (1465–1704)	6.38	–	–
August	1133 (1050–1224)	7.32	1710 (1585–1842)	6.17	1474 (1365–1591)	6.53	–	–
September	1094 (1015–1180)	7.43	1471 (1363–1586)	6.91	1427 (1323–1539)	6.68	–	–
October	1146 (1063–1234)	7.51	1433 (1329–1545)	7.16	1442 (1337–1555)	6.78	–	–
November	1217 (1130–1310)	7.85	1395 (1293–1503)	7.09	1442 (1336–1555)	6.90	–	–
December	1203 (1115–1299)	5.13	1542 (1430–1661)	6.57	1846 (1713–1993)	5.51	–	–
Total	9576 (8877–10,329)	7.45	16,104 (14,930–17,358)	6.91	18,195 (16,899–19,552)	6.54	2,944 (2726–3176)	5.77

January 2019-February 2020—pre-pandemic period; March 2020-February 2022—pandemic period

*ED* Emergency Department

*Diagnostic Groups—Suicidality, Eating Disorder, Mania, Psychosis, Substance Use

^Patients who were identified as presenting for multiple diagnoses were counted per diagnostic groups

**Table 2:** Se and Sp for Mental Health Presentation Estimates

Mental health diagnostic groups	Sensitivity *(Se)*	Specificity *(Sp)*
Suicidality	0.97	0.99
Eating disorder	0.99	0.99
Mania	0.86	0.95
Psychosis	0.99	0.99
Substance use	0.88	0.89

### Trends in presentation rates over the COVID-19 pandemic

Whilst there is a general upward trend in the overall number of presentations across all five diagnostic groups, there is evidence of peaks and troughs throughout the pandemic period (Fig. 1). Notable troughs in the overall number of presentations occur in April 2020 (*N* = 997), April 2021 (*N* = 1382) and August 2021 (*N* = 1474). There was a clear peak in the presentation numbers occurring in August 2020 (*N* = 1710). In the time periods before and during the pandemic, there were recurring peaks at the end and beginning of each year: November 2019 (*N* = 1217), December 2020 (*N* = 1542), January 2021 (*N* = 1603), December 2021 (*N* = 1846), and January 2022 (*N* = 1742).

### Presentation rates per diagnosis

**Fig. 2:**
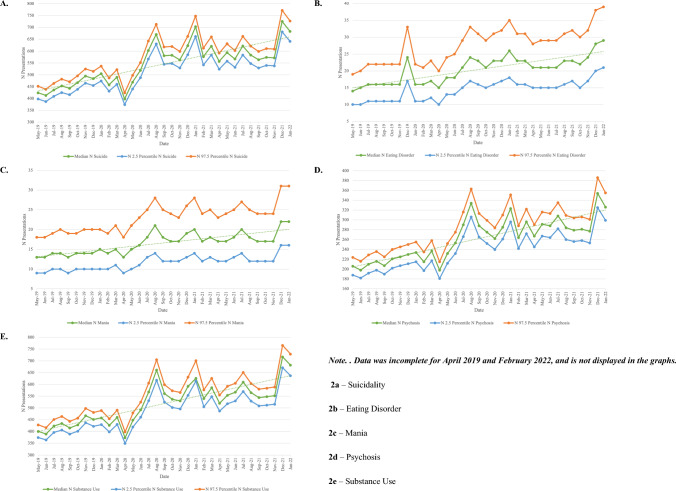
Bayesian Estimate (with 95% probability interval) of Presentations Made to Gold Coast EDs Across the Five Diagnostic Groups Between April 2019 and February 2022 Per Diagnostic Group

Across the study period, suicidality (*N* = 18,746), substance use (*N* = 17,809) and psychosis (*N* = 8,994) were the most prevalent mental health presentations to the ED. Figure 2 displays presentations per diagnostic group (2a—suicidality, 2b—eating disorder, 2c—mania, 2d—psychosis, 2e—substance use). Further tables displaying the numbers of presentation per month for each diagnostic group are provided in Table 3 (suicidality), Table 4 (eating disorder), Table 5 (mania), Table 6 (psychosis), and Table 7 (substance use).

**Table 3:** Suicidal Presentations to the Gold Coast Emergency Departments, April 2019—February 2022

Month	2019		2020		2021		2022	
	Suicidality ED presentations *N* (95% CI)	% Of all ED presentations	Suicidality ED presentations *N* (95% CI)	% of all ED presentations	Suicidality ED presentations *N* (95% CI)	% Of all ED presentations	Suicidality ED presentations *N* (95% CI)	% Of all ED presentations
January	–	–	505 (474–537)	3.17	703 (661–748)	2.61	683 (641–727)	2.02
February	–	–	458 (431–487)	3.1	577 (542–613)	2.78	486 (456–516)	2.83
March	–	–	490 (460–522)	2.82	621 (584–660)	2.75	–	–
April	240 (225–256)	2.96	398 (374–424)	2.86	557 (524–592)	2.81	–	–
May	424 (398–452)	2.93	469 (440–498)	2.78	594 (558–631)	2.82	–	–
June	413 (387–439)	2.93	519 (488–552)	2.7	567 (532–603)	2.52	–	–
July	436 (409–464)	2.88	603 (567–642)	2.64	622 (584–662)	2.51	–	–
August	453 (425–482)	2.92	670 (630–713)	2.42	584 (548–622)	2.59	–	–
September	443 (416–471)	3.01	581 (545–618)	2.73	564 (529–599)	2.64	–	–
October	467 (439–496)	3.07	583 (548–620)	2.91	574 (540–611)	2.7	–	–
November	495 (465–525)	3.19	563 (529–599)	2.86	572 (538–609)	2.74	–	–
December	484 (455–515)	3.08	623 (585–662)	2.65	725 (681–772)	2.16	–	–
Total	3855 (3619–4100)	3.00	6462 (6071–6874)	2.77	7260 (6821–7722)	2.61	1169 (1097–1243)	2.29

January 2019-February 2020—pre-pandemic period; March 2020-February 2022—pandemic period

*ED* Emergency Department, *CI* Confidence Interval

**Table 4:** Eating Disorder Presentations to the Gold Coast Emergency Departments, April 2019—February 2022

Month	2019		2020		2021		2022	
	Eating disorder ED presentations *N* (95% CI)	% of all ED presentations	Eating disorder ED presentations *N* (95% CI)	% Of all ED presentations	Eating disorder ED presentations *N* (95% CI)	% Of all ED presentations	Eating disorder ED presentations *N* (95% CI)	% Of all ED presentations
January	–	–	16 (11–22)	0.1	26 (18–35)	0.1	29 (21–39)	0.09
February	–	–	16 (11–21)	0.11	23 (16–31)	0.11	19 (13–25)	0.11
March	–	–	17 (12–23)	0.1	23 (16–31)	0.1	–	–
April	8 (6–11)	0.1	15 (10–20)	0.11	21 (15–28)	0.11	–	–
May	14 (10–19)	0.1	18 (13–24)	0.11	21 (15–29)	0.1	–	–
June	15 (10–20)	0.11	18 (13–25)	0.09	21 (15–29)	0.09	–	–
July	16 (11–22)	0.11	21 (15–29)	0.09	21 (15–29)	0.08	–	–
August	16 (11–22)	0.1	24 (17–33)	0.09	23 (16–31)	0.1	–	–
September	16 (11–22)	0.11	23 (16–31)	0.11	23 (17–32)	0.11	–	–
October	16 (11–22)	0.1	21 (15–29)	0.1	22 (15–30)	0.1	–	–
November	16 (11–22)	0.1	23 (16–31)	0.12	24 (17–32)	0.11	–	–
December	24 (17–33)	0.1	23 (17–32)	0.1	28 (20–38)	0.08	–	–
Total	141 (98–193)	0.11	235 (166–320)	0.10	276 (195–375)	0.10	48 (34–64)	0.09

January 2019-February 2020—pre-pandemic period; March 2020-February 2022—pandemic period

*ED* Emergency Department, *CI* Confidence Interval

**Table 5:** Mania Presentations to the Gold Coast Emergency Departments, April 2019—February 2022

Month	2019		2020		2021		2022	
	Mania ED presentations *N* (95% CI)	% Of all ED presentations	Mania ED presentations *N* (95% CI)	% Of all ED presentations	Mania ED presentations *N* (95% CI)	% Of all ED presentations	Mania ED presentations *N* (95% CI)	% Of all ED presentations
January	–	–	15 (10–20)	0.09	20 (14–28)	0.07	22 (16–31)	0.07
February	–	–	14 (10–19)	0.09	17 (12–24)	0.08	15 (10–21)	0.09
March	–	–	15 (11–21)	0.09	18 (13–25)	0.08	–	–
April	8 (6–11)	0.1	13 (9–18)	0.09	17 (12–23)	0.09	–	–
May	13 (9–18)	0.09	15 (10–21)	0.09	17 (12–24)	0.08	–	–
June	13 (9–18)	0.09	16 (11–23)	0.08	18 (13–25)	0.08	–	–
July	14 (10–19)	0.09	18 (13–25)	0.08	20 (14–27)	0.08	–	–
August	14 (10–20)	0.09	21 (14–28)	0.08	18 (12–25)	0.08	–	–
September	13 (9–19)	0.09	18 (12–25)	0.08	17 (12–24)	0.08	–	–
October	14 (10–19)	0.09	17 (12–24)	0.08	17 (12–24)	0.08	–	–
November	14 (10–20)	0.09	17 (12–23)	0.09	17 (12–24)	0.08	–	–
December	14 (10–20)	0.09	19 (13–26)	0.08	22 (16–31)	0.07	–	–
Total	117 (83–164)	0.14	198 (137–273)	0.08	218 (154–304)	0.08	37 (26–52)	0.07

January 2019-February 2020—pre-pandemic period; March 2020-February 2022—pandemic period

*ED* Emergency Department, *CI* Confidence Interval

**Table 6:** Psychosis Presentations to the Gold Coast Emergency Departments, April 2019—February 2022

Month	2019		2020		2021		2022	
	Psychosis ED presentations *N* (95% CI)	% Of all ED presentations	Psychosis ED presentations *N* (95% CI)	% Of all ED presentations	Psychosis ED presentations *N* (95% CI)	% Of all ED presentations	Psychosis ED presentations *N* (95% CI)	% Of all ED presentations
January	–	–	234 215–255)	1.47	323 (296–351)	1.2	326 (299–355)	0.96
February	–	–	215 (197–235)	1.46	264 (242–288)	1.27	226 (207–246)	1.32
March	–	–	237 (217–258)	1.37	296 (272–322)	1.31	–	–
April	114 (104–125)	1.41	198 (181–215)	1.42	267 (245–290)	1.35	–	–
May	206 (188–224)	1.42	232 (212–252)	1.38	291 (267–316)	1.38	–	–
June	198 (182–216)	1.4	253 (232–275)	1.31	288 (264–313)	1.28	–	–
July	210 (1292–229)	1.39	290 (266–316)	1.27	308 (282–335)	1.24	–	–
August	216 (198–236)	1.39	334 (306–363)	1.2	284 (360–309)	1.26	–	–
September	207 (190–225)	1.41	288 (265–313)	1.35	279 (256–304)	1.31	–	–
October	221 (202–240)	1.45	275 (252–299)	1.37	281 (258–306)	1.32	–	–
November	225 (207–245)	1.45	262 (240–284)	1.33	277 (253–301)	1.33	–	–
December	230 (211–250)	1.47	285 (261–310)	1.21	354 (325–386)	1.06	–	–
Total	1827 (1674–1990)	1.42	3103 (2844–3375)	1.33	3512 (3220–3821)	1.26	552 (506–601)	1.08

January 2019-February 2020—pre-pandemic period; March 2020-February 2022—pandemic period

*ED* Emergency Department, *CI* Confidence Interval

**Table 7:** Substance Use Presentations to the Gold Coast Emergency Departments, April 2019—February 2022

Month	2019		2020		2021		2022	
	Substance use ED presentations *N* (95% CI)	% Of all ED presentations	Substance use ED presentations *N* (95% CI)	% Of all ED presentations	Substance use ED presentations *N* (95% CI)	% Of all ED presentations	Substance use ED presentations *N* (95% CI)	% Of all ED presentations
January	–	–	458 (429–489)	2.87	626 (614–701)	2.44	682 (637–729)	2.02
February	–	–	426 (398–454)	2.88	540 (505–577)	2.6	456 (426–487)	2.66
March	–	–	460 (430–491)	2.65	586 (548–626)	2.59	–	–
April	229 (214–244)	2.83	373 (349–398)	2.68	520 (487–555)	2.62	–	–
May	400 (374–428)	2.76	448 (419–479)	2.66	554 (518–592)	2.63	–	–
June	389 (364–416)	2.76	492 (461–525)	2.56	567 (530–605)	2.52	–	–
July	423 (396–451)	2.79	568 (531–606)	2.48	610 (570–651)	2.46	–	–
August	434 (406–464)	2.8	661 (618–705)	2.38	565 (529–604)	2.5	–	–
September	415 (389–443)	2.82	561 (525–599)	2.63	544 (509–580)	2.55	–	–
October	428 (401–457)	2.81	537 (502–573)	2.68	548 (512–584)	2.58	–	–
November	467 (437–498)	3.01	530 (496–566)	2.7	552 (516–589)	2.64	–	–
December	451 (422–481)	2.87	592 (554–631)	2.53	717 (671–766)	2.14	–	–
Total	3636 (3403–3882)	2.83	6106 (5712–6516)	2.62	6929 (6509–7430)	2.49	1138 (1063–1216)	2.23

January 2019-February 2020—pre-pandemic period; March 2020-February 2022—pandemic period

*ED* Emergency Department, *CI* Confidence Interval

### Rates of presentations for mental health relative to other emergency department presentations

**Fig. 3:**
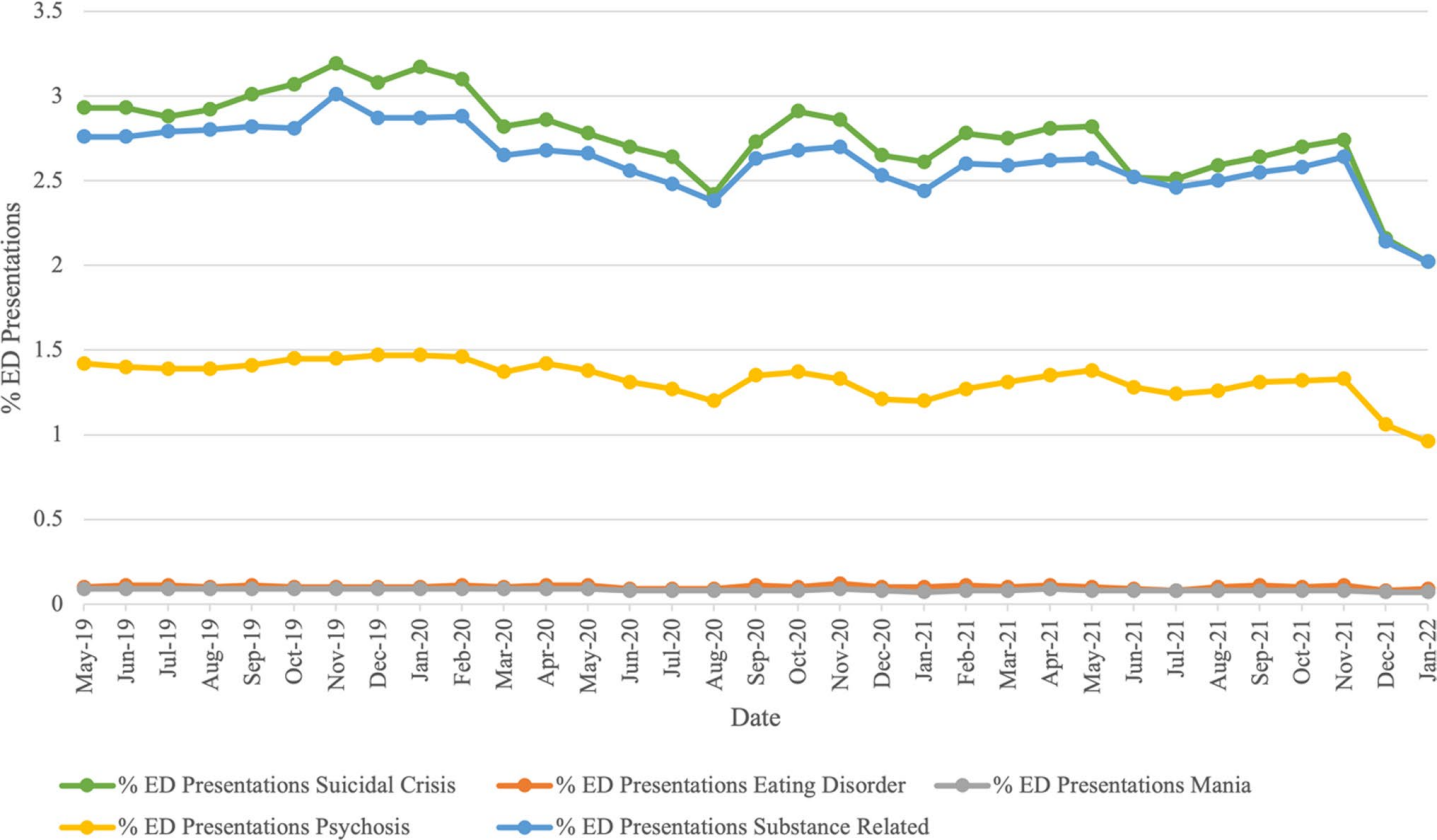
Percentage of Mental Health Presentations Relative to Total ED Presentations (Note. Percentages for each presentation can be viewed in the Tables 3–7. Data was incomplete for April 2019 and February 2022, and is not displayed in the graph.)

Figure 3 shows the number of mental health presentations relative to the total number of ED presentations. Relative rates of mental health presentations can be seen for all five diagnostic groups in Table 1, and per diagnostic group in Table 3 (suicidality), Table 4 (eating disorder), Table 5 (mania), Table 6 (psychosis), and Table 7 (substance use). Although the overall number of mental health presentations increased across the study period, the proportion of these presentations, relative to the total number of ED presentations (prevalence), decreased. At the beginning of the study period (April 2019), presentations across the diagnostic groups examined accounted for 7.40%, compared to 7.00% of all ED presentations at the end of the study period (February 2022). This decrease was evident for suicidality (2.96% to 2.85%), mania (0.10% to 0.09%), psychosis (1.41% to 1.32%), and substance use (2.83% to 2.66%). Prevalence of eating disorders remained relatively stable (0.10% to 0.11%).

## Discussion

This study analysed the trends that occurred before and during the COVID-19 pandemic in ED presentations across five mental health diagnostic groups. This revealed an increasing trend in the number of mental health presentations across the COVID-19 pandemic, which was consistent across all diagnostic groups examined. Decreases were found earlier in the pandemic, when lockdown restrictions were in place, with increases consistent with the times at which public health restrictions were lifted (refer to timeline in Appendix C). However, the proportion of these presentations, relative to the total number of ED presentations, decreased (7.40% in April 2019 vs. 7.00% Feb 2022).

The COVID-19 pandemic significantly impacted mental health, with disruptions to social interaction and other life events, changes in public health directions, and a high level of psychological distress [1, 30]. Whilst not as restricted as other regions in Australia, the densely population Southeast Queensland, including the Gold Coast was highly affected by lockdowns and other restrictions which were imposed on Queensland. Public health restrictions during this time reduced accessibility to in-person mental health care services, whilst increasing the prevalence of mental health symptoms [31]. It is possible that due to this, more patients were accessing the ED, which remained available during the pandemic, to access mental health support that they would have otherwise received within the community [32].

Both increases and decreases were observed at certain times throughout the pandemic (i.e., decreases in April 2020, April 2021, and August 2021 and an increase in August 2020), which coincided with key COVID-19 pandemic events (timeline provided in Appendix C), with the decreases in cases consistent with more restrictive COVID-19 events (i.e., state lockdowns and restrictions), and increases at times where lockdown and other public health restrictions were lifted. Possibly in months where COVID-19 presentation rates were high, there was an increased perceived risk in attending the ED for mental health care. This risk may be reduced when restrictions were eased, with a perceived increase in safety relating to risk of infection, resulting in an increase in mental health presentations.

Mental health presentations consistently increased in November, December, and January. This was not only during the COVID-19 pandemic, but in 2019 (pre-pandemic), which suggests that the holiday period corresponds to heightened psychological distress. Christmas-related distress has been examined previously, such as by Velamoor et al. [33] who found that the majority expressed negative feelings, citing financial burden, loneliness, and expectations from others as reasons for distress during the Christmas period [33]. The Gold Coast hosts a post-graduation social event for school leavers in in November and December each year (‘Schoolies’) and sees a significant rise in alcohol and substance consumption, incidences of related harm, and is likely to be related to increases in presentations during this time [34].

It was also found that there was a decrease in ED presentations, relative to the total number of ED presentations, for suicidality, mania, psychosis, and substance use in 2022 compared to 2019. Dragovic et al. [4] similarly found a 26% decrease in suicidal presentations to EDs in Western Australia compared to the previous year. Likewise, the relative decrease in mania-related presentations is similar to Seifert et al. [8], who found a decrease in ED presentations related to affective disorders in Germany compared to the previous year.

In contrast, the trends in presentations for the other diagnostic groups (psychosis, substance use, and eating disorders) remains unclear. In Germany, Seifert et al. [8] did not find any differences in the number of presentations for substance use and psychosis in 2019 compared to 2020. In Western Australia, Haripersad et al. [35] found admissions for anorexia nervosa increased during the COVID-19 pandemic, consistent with similar findings in other countries [36]. This contrasts with the findings of our study, which found that there were fewer presentations of eating disorders, whilst the number of relative presentations remained relatively stable, however, this does not necessarily suggest that there is a lower prevalence rate; rather that these presentations are more likely to seek support from services besides the ED.

This work’s secondary contribution is in the MEDREADS algorithm’s description. The MEDREADS framework was used to collect data, including triage notes, to identify mental health presentations. The need for and value of such approaches in Australian EDs has been highlighted in prior research conducted in Queensland and Victoria [20, 21]. Their improvement supports measurement for both research and more direct healthcare strategies. In the case of MEDREADS, three elements may allow for further improvement of the underlying models. First, the models do not allow for interactions where the presence of multiple variables simultaneously may affect risk beyond their individual contributions. Second, the risk scores produced by the algorithms cannot be interpreted other than by comparison to a specific threshold value. As produced by alternative techniques, probabilistic predictions allow for more flexible use of models. Third, several of the variables used in the model are semantically equivalent but can be associated with different risk scores. For example, different risk scores are assigned depending on whether the triage notes contain the string “drown himself” or “drown himself”. Variation in risk scores for semantically equivalent information is undesirable as it decreases model reliability and undermines the confidence of those using the model. Finally, the impact of COVID-19 on mental health presentations to ED is likely to be a result of a number of factors that differ across health services and countries, such as the severity of COVID-19 cases, existing health infrastructure, governance structures, and population characteristics [3].

## Clinical implications

The main implications of our findings are twofold. Firstly, our study contributes to the understanding of the trends in mental health presentations to the Gold Coast EDs during the COVID-19 pandemic. By using a novel machine learning (ML) approach, we were able to analyse a large volume of mental health data and identify patterns and trends that would have been challenging to uncover using traditional methods (manually coding). This provides important insights into the impact of the pandemic on mental health and helps healthcare professionals and policymakers make informed decisions regarding resource allocation, intervention strategies, and support services, particularly with regards to specific health conditions which may require more specialised care. This work can also be used to inform planning and resource allocation in the event of future major health events, such as future health pandemics.

Secondly, our ML algorithms have demonstrated their effectiveness in analysing mental health data derived from hospital EDs. While other populations may have shown different patterns of mental health presentations, our approach has proven to be a valuable tool in understanding the unique dynamics of the Gold Coast population during the pandemic. The use of ML techniques offers the potential for scalability and transferability to other healthcare settings, enabling similar analyses to be conducted in different regions or during different time periods.

We acknowledge that the findings are specific to the Gold Coast population and the unique circumstances of the COVID-19 pandemic. The Gold Coast is a unique, transient population, with many interstate and international visitors each year. Factors such as demographic characteristics, cultural context, healthcare infrastructure, and pandemic-related restrictions may influence the generalisability of our results to other regions. Therefore, caution should be exercised when extrapolating our findings to different populations. Whilst the findings themselves are unique to the Gold Coast population, the MEDREADS algorithm has potential to be used across other settings, such as other EDs, to examine patterns in mental health presentations across the five diagnostic groupings assessed in this research. Future research could apply the MEDREADS algorithm to other EDs across Australia, to compare such presentations across the same time period. It is also important to consider that the presentations made in this study may not be standalone, rather, it is likely that the same individual may have made multiple presentations across the study period. Assessing re-presentations to the ED for mental health is important, and will be examined in a broader follow-up study.

## Conclusions

This study examines the trends in mental health presentations made to the Gold Coast EDs during the COVID-19 pandemic, using a novel ML approach. Whilst other populations were in consistent with the rates of presentations within the Gold Coast, the ML algorithms that we have presented are an effective method of analysing mental health data derived from the hospital ED. Future research should consider the potential improvement of the models used to identify relevant presentations. Such improvement could be driven by considering variable interactions, enforcing consistency in risk scores assigned to semantically equivalent text, and assessing alternative, probabilistic models.

### Electronic supplementary material

Below is the link to the electronic supplementary material.

Supplementary file1 (DOCX 223 KB)

## Data Availability

Due to the sensitivity of the data in this research, the data is not available to be shared.

## Appendix A

Variables Used to Develop MEDREADS

**Table:**

MEDREADS variables	*N* Values
Mode of arrival	12
Australasian triage scale (ATS)	5
Consultant speciality description	2
Consultation type	13
Departure destination description	12
Admission specialty	7
Diagnosis (primary diagnosis, additional diagnosis one, additional diagnosis two)	109
Presenting problem description	3
Triage text	68

## Appendix B

Defined Values for Cerner FirstNet® Variables Included in MEDREADS

**Table:**

Variable	Defined values
Mode of arrival	Ambulance (road—paramedic)
	Walked in/public or private transport
	Ambulance & police EEA
	Ambulance under EEA
	Police or prison vehicle
	Police under EEA
	Ambulance (road—pt transport officer)
	Ambulance (helicopter)
	Community services
	Other
	Ambulance (fixed wing)
	Ambulance (road)
Australasian triage scale	1—Life threatening conditions
	2—Imminently life threatening, time sensitive treatment needed, or severe pain
	3—Potentially life threatening, situational urgency, or severe pain
	4—Potentially serious condition, situational urgency, or complex case
	5—Less urgent or clinical-administrative problems
Consultant speciality description	Psychiatry
	Psychology
Consultation type	Consult to mental health
	Consult to psychiatry
	Consult to emergency psychiatric service
	Follow up to social work
	Consult to drug and alcohol brief interv
	Consult to alcohol and drug assessment U
	Consult order
	Consult to homeless liaison officer
	Consult to child and youth mental health
	Consult to psychology
	Consult to indigenous mental health work
	Consult to clozapine coordinator
	Consult to neuropsychology
Departure destination description	GAMH1
	GAMH2
	GAMH3
	GAMH4
	GAMH5
	GAMH6
	GAMH7
	GAMH8
	RYAMH
	RCYMH
	RAAMH
	RTMH
Admission specialty	Psychiatry
	Child and Youth Mental Health (CYMHS)
	Psychology
	Psychiatric Adult Residential
	Emergency Psychiatric Service
	Psychogeriatric
	Drug and Alcohol Brief Intervention Team
Diagnosis (primary diagnosis, additional diagnosis one, additional diagnosis two)	Acute drug overdose
	Antiallergenic drug overdose
	Anticholinergic drug overdose
	Aspirin overdose
	At risk for deliberate self-harm
	At risk for suicide
	At risk of DSH—deliberate self-harm
	Attempted suicide—hanging
	Benzodiazepine overdose
	Beta-adrenoceptor agonist overdose
	Beta-blocker overdose
	Carbon monoxide poisoning
	Clonazepam overdose
	Clonidine overdose
	Cuts self
	Cutting own wrists
	Deliberate self-harm
	Deliberate self-cutting
	Deliberate self-harm
	Diazepam overdose
	Diazepam overdose of undetermined intent
	Drug overdose
	Drug overdose—suicide
	Drug overdose of undetermined intent
	Feeling suicidal
	Fluoxetine overdose
	Found hanging self
	Hanging self
	Heroin overdose
	High suicide risk
	Ibuprofen overdose
	Injury due to suicide attempt
	Insulin overdose
	Intent of deliberate self-harm with detailed plans
	Intentional antihypertensive overdose
	Intentional aspirin overdose
	Intentional benzodiazepine overdose
	Intentional diazepam overdose
	Intentional drug overdose
	Intentional drug overdose by tablet
	Intentional fluoxetine overdose
	Intentional heroin overdose
	Intentional insulin overdose
	Intentional mefenamic acid overdose
	Intentional naproxen overdose
	Intentional non-opiate analgesic overdose
	Intentional opiate analgesic overdose
	Intentional osmotic diuretic overdose
	Intentional overdose of tricyclic antidepressant
	Intentional paracetamol overdose
	Intentional paracetamol poisoning
	Intentional sertraline overdose
	Intentional temazepam overdose
	Intentional venlafaxine overdose
	Intentionally harming self
	Lithium overdose
	Low suicide risk
	Moderate suicide risk
	Multiple superficial injuries of forearm
	NSAID—Non-steroidal anti-inflammatory overdose
	OD—Overdose of drug
	Overdose
	Overdose of angiotensin-converting-enzyme inhibitors
	Overdose of antidepressant drug
	Overdose of benzodiazepine of undetermined intent
	Overdose of cocaine
	Overdose of drug groups primarily affecting the central nervous system
	Overdose of drug primarily affecting the respiratory system
	Overdose of illicit drug
	Overdose of opiate
	Overdose of sodium valproate
	Overdose of temazepam
	Overdose of tricyclic antidepressant
	Oxazepam overdose
	Paracetamol overdose
	Paracetamol overdose of undetermined intent
	Paracetamol poisoning
	Planning suicide
	Polypharmacy overdose
	Propranolol overdose
	Self-inflicted injury
	Self-inflicted lacerations to wrist
	Self-poisoning by carbon monoxide
	Self-poisoning by plants or parts of Plants
	Self-electrocution
	Self-poisoning by carbon monoxide
	Self-harm
	Self-strangulation
	Sertraline overdose
	Stimulant overdose
	Strangulation
	Suicidal
	Suicidal behaviour
	Suicidal deliberate poisoning
	Suicidal ideation
	Suicidal intent
	Suicidal plans
	Suicidal thoughts
	Suicide
	Suicide attempt
	Suicide gesture
	Suicide risk
	Thoughts of deliberate self-harm
	Thoughts of self-harm
	Threatening suicide
	Tricyclic antidepressant poisoning
	Unsuccessful suicide attempt
	Venlafaxine overdose
	Warfarin overdose
	Zolpidem overdose
Presenting problem description	Suicidal-homicidal ideation
	Overdose/toxic exposure
	Strangulation/asphyxia incl hanging
Triage text	Suicid
	Inten
	Ideation
	Threaten
	Self harm
	Selfharm
	Self-harm
	Harm self
	Harm himself
	Harm herself
	Harm him self
	Harm her self
	Denies
	States
	States wants to
	Stating
	Attempt
	End it
	End life
	End his life
	End her life
	Plan
	With plan
	With intent
	Deliberat
	Dsh
	Be dead
	Life
	Thought
	Found
	O/d
	Od
	Overdose
	Polysubstance
	Poly substance
	Polypharmacy
	Poly pharmacy
	Ingest
	kill self
	kill himself
	kill herself
	kill her self
	kill him self
	Not want to be here
	Wants to die
	Self inflicted
	Selfinflicted
	Self-inflicted
	Jump off
	Jumped off
	Cut
	Cut wrist
	Drown himself
	Drown herself
	Drown him self
	Drown her self
	Immers
	Hang
	Hung
	Gas himself
	Gas herself
	Gas him self
	Gas her self
	Carbon mon
	Lacerat
	Strang
	Si
	Superficial

## Appendix C

Timeline of Key COVID-19 Pandemic Events Occurring in Queensland, Australia

**Table:**

Date	Event
March 2020	COVID-19 is declared a global pandemic by the World Health Organisation
	Australia announces lockdowns and restrictions to be implemented nationwide
	Australia closes its borders to all international travellers. Non-citizens and -residents are unable to exit Australia, and citizens and residents are unable to leave without exemption
April 2020	Stage 4 restrictions are implemented in Queensland, the most restrictive stage since the start of the COVID-19 pandemic
	All individuals who have been in a declared COVID-19 hotspot within the past 14 days must self-quarantine for 14 days
	Entry into Queensland is restricted. All individuals require a permit to enter the Queensland border
May 2020	Queensland enters Stage 1 in the roadmap to end COVID-19 restrictions
June 2020	Queensland enters Stage 2 in the roadmap to end COVID-19 restrictions
July 2020	Queensland enters Stage 3 in the roadmap to end COVID-19 restrictions
January 2021	Greater Brisbane enters a three-day lockdown
February 2021	Queensland opens its borders to all Australian states and territories except for Western Australia
	The first COVID-19 vaccination in Queensland is administered at the Gold Coast University Hospital
	The Check in QLD app is launched to assist with contact tracing efforts
March 2021	Greater Brisbane enters a three-day lockdown
April 2021	Restrictions are introduced in Queensland, including mask mandates, visitor restriction, and venue capacity limits
June 2021	Masks become mandatory as a case is identified in Southeast Queensland as being infected with the Delta variant
	Lockdown is announced for Southeast Queensland, Townsville, Magnetic Island, and Palm Island
July 2021	Anti-lockdown protests take place in Brisbane
	Southeast Queensland enters a 3-day lockdown following identified cases of the Delta variant that were locally acquired
August 2021	The Southeast Queensland lockdown is extended as more cases are identified
	Cairns enters a 3-day lockdown
December 2021	Vaccination against COVID-19 becomes mandatory for all individuals (unless medically exempted) for many public places. Restrictions for unvaccinated individuals are implemented
	Masks become mandatory across Queensland
January 2022	A rapid increase in cases is observed, with Omicron being the most prevalent variant
	Queensland opens its borders to all domestic travellers
February 2022	Australia opens its international borders to all vaccinated travellers
