# Supplementary material for: Using machine learning to mine mental health diagnostic groups from emergency department presentations before and during the COVID-19 pandemic
Source: Discov Ment Health. 2023 Nov 6;3(1):22. doi: 10.1007/s44192-023-00047-0 (PMC10628018; doi:10.1007/s44192-023-00047-0)
Supplement: Supplementary file 1 — Supplementary file1 (DOCX 223 KB) [file 44192_2023_47_MOESM1_ESM.docx]

> heet1") firstrow

(153 vars, 39,885 obs)

. roctab EDOCase MEDREADS_EDO_Score, graph summary title(EDO Cases) detail

Detailed report of sensitivity and specificity

------------------------------------------------------------------------------

Correctly

Cutpoint Sensitivity Specificity classified LR+ LR-

------------------------------------------------------------------------------

( >= -7 ) 100.00% 0.00% 0.11% 1.0000

( >= -6 ) 100.00% 0.16% 0.27% 1.0016 0.0000

( >= -5 ) 100.00% 57.93% 57.97% 2.3769 0.0000

( >= -4 ) 100.00% 58.29% 58.33% 2.3973 0.0000

( >= -3 ) 100.00% 58.30% 58.34% 2.3979 0.0000

( >= -2 ) 100.00% 77.20% 77.23% 4.3863 0.0000

( >= -1 ) 100.00% 77.32% 77.35% 4.4096 0.0000

( >= 0 ) 100.00% 77.81% 77.83% 4.5059 0.0000

( >= 1 ) 100.00% 79.52% 79.55% 4.8837 0.0000

( >= 2 ) 100.00% 80.94% 80.97% 5.2478 0.0000

( >= 3 ) 100.00% 82.68% 82.70% 5.7741 0.0000

( >= 4 ) 100.00% 82.86% 82.88% 5.8349 0.0000

( >= 5 ) 100.00% 84.15% 84.17% 6.3099 0.0000

( >= 6 ) 100.00% 84.41% 84.43% 6.4146 0.0000

( >= 7 ) 100.00% 85.13% 85.14% 6.7242 0.0000

( >= 8 ) 100.00% 85.87% 85.88% 7.0753 0.0000

( >= 9 ) 100.00% 88.73% 88.75% 8.8753 0.0000

( >= 10 ) 100.00% 88.98% 89.00% 9.0775 0.0000

( >= 11 ) 100.00% 89.57% 89.59% 9.5910 0.0000

( >= 12 ) 100.00% 92.93% 92.94% 14.1481 0.0000

( >= 13 ) 100.00% 92.98% 92.99% 14.2442 0.0000

( >= 14 ) 100.00% 93.94% 93.95% 16.4973 0.0000

( >= 15 ) 100.00% 96.03% 96.03% 25.1680 0.0000

( >= 16 ) 100.00% 96.22% 96.23% 26.4900 0.0000

( >= 17 ) 100.00% 96.78% 96.79% 31.0772 0.0000

( >= 18 ) 100.00% 97.08% 97.08% 34.2277 0.0000

( >= 19 ) 100.00% 97.38% 97.38% 38.1254 0.0000

( >= 20 ) 100.00% 97.63% 97.64% 42.2493 0.0000

( >= 21 ) 100.00% 97.89% 97.89% 47.3733 0.0000

( >= 22 ) 100.00% 98.03% 98.03% 50.8175 0.0000

( >= 23 ) 100.00% 98.09% 98.09% 52.4225 0.0000

( >= 24 ) 100.00% 98.26% 98.26% 57.4907 0.0000

( >= 25 ) 100.00% 98.32% 98.32% 59.3755 0.0000

( >= 26 ) 100.00% 98.35% 98.35% 60.6408 0.0000

( >= 27 ) 100.00% 98.43% 98.43% 63.5424 0.0000

( >= 28 ) 100.00% 98.77% 98.77% 81.3082 0.0000

( >= 29 ) 100.00% 98.82% 98.82% 84.4090 0.0000

( >= 30 ) 100.00% 98.84% 98.84% 86.2361 0.0000

( >= 31 ) 100.00% 99.01% 99.01% 101.3767 0.0000

( >= 32 ) 100.00% 99.05% 99.05% 104.8442 0.0000

( >= 33 ) 100.00% 99.08% 99.08% 109.1539 0.0000

( >= 34 ) 100.00% 99.17% 99.17% 120.3655 0.0000

( >= 35 ) 100.00% 99.23% 99.23% 129.7755 0.0000

( >= 36 ) 100.00% 99.32% 99.32% 146.4753 0.0000

( >= 37 ) 100.00% 99.36% 99.36% 156.2390 0.0000

( >= 38 ) 100.00% 99.41% 99.41% 169.5364 0.0000

( >= 39 ) 100.00% 99.46% 99.46% 183.5974 0.0000

( >= 40 ) 100.00% 99.51% 99.51% 202.2373 0.0000

( >= 41 ) 100.00% 99.55% 99.55% 220.1152 0.0000

( >= 42 ) 100.00% 99.58% 99.58% 240.0059 0.0000

( >= 43 ) 100.00% 99.64% 99.64% 274.7668 0.0000

( >= 44 ) 100.00% 99.66% 99.66% 292.9507 0.0000

( >= 45 ) 100.00% 99.69% 99.69% 323.9065 0.0000

( >= 46 ) 97.73% 99.73% 99.73% 360.5209 0.0228

( >= 47 ) 97.73% 99.75% 99.74% 385.4974 0.0228

( >= 48 ) 97.73% 99.77% 99.77% 427.8612 0.0228

( >= 49 ) 97.73% 99.80% 99.79% 480.6856 0.0228

( >= 50 ) 97.73% 99.81% 99.81% 519.1420 0.0228

( >= 51 ) 97.73% 99.82% 99.82% 540.7510 0.0228

( >= 52 ) 97.73% 99.83% 99.83% 572.5854 0.0228

( >= 53 ) 93.18% 99.83% 99.83% 562.4984 0.0683

( >= 54 ) 90.91% 99.84% 99.83% 565.9291 0.0911

( >= 55 ) 90.91% 99.85% 99.84% 603.6596 0.0910

( >= 56 ) 90.91% 99.86% 99.85% 646.7805 0.0910

( >= 57 ) 88.64% 99.88% 99.86% 720.7038 0.1138

( >= 58 ) 88.64% 99.89% 99.88% 840.8298 0.1138

( >= 59 ) 88.64% 99.90% 99.89% 905.5140 0.1137

( >= 60 ) 88.64% 99.92% 99.90% 1.1e+03 0.1137

( >= 61 ) 86.36% 99.92% 99.90% 1.0e+03 0.1365

( >= 63 ) 86.36% 99.92% 99.91% 1.1e+03 0.1365

( >= 64 ) 86.36% 99.93% 99.92% 1.3e+03 0.1365

( >= 65 ) 86.36% 99.94% 99.93% 1.5e+03 0.1364

( >= 66 ) 86.36% 99.95% 99.94% 1.8e+03 0.1364

( >= 67 ) 84.09% 99.96% 99.94% 2.2e+03 0.1592

( >= 68 ) 84.09% 99.96% 99.95% 2.4e+03 0.1591

( >= 72 ) 84.09% 99.97% 99.95% 2.8e+03 0.1591

( >= 73 ) 84.09% 99.97% 99.95% 3.0e+03 0.1591

( >= 75 ) 84.09% 99.97% 99.96% 3.4e+03 0.1591

( >= 77 ) 84.09% 99.98% 99.96% 3.7e+03 0.1591

( >= 78 ) 84.09% 99.98% 99.96% 4.2e+03 0.1591

( >= 79 ) 79.55% 99.98% 99.96% 4.5e+03 0.2046

( >= 80 ) 79.55% 99.98% 99.96% 5.3e+03 0.2046

( >= 82 ) 77.27% 99.99% 99.96% 6.2e+03 0.2273

( >= 85 ) 77.27% 99.99% 99.96% 7.7e+03 0.2273

( >= 86 ) 75.00% 99.99% 99.96% 7.5e+03 0.2500

( >= 96 ) 72.73% 99.99% 99.96% 7.2e+03 0.2728

( >= 97 ) 68.18% 99.99% 99.95% 6.8e+03 0.3182

( >= 101 ) 68.18% 99.99% 99.96% 9.1e+03 0.3182

( >= 104 ) 63.64% 99.99% 99.95% 8.5e+03 0.3637

( >= 106 ) 59.09% 99.99% 99.95% 1.2e+04 0.4091

( >= 107 ) 56.82% 99.99% 99.95% 1.1e+04 0.4318

( >= 109 ) 54.55% 99.99% 99.94% 1.1e+04 0.4546

( >= 111 ) 52.27% 99.99% 99.94% 1.0e+04 0.4773

( >= 112 ) 50.00% 99.99% 99.94% 1.0e+04 0.5000

( >= 114 ) 50.00% 100.00% 99.94% 2.0e+04 0.5000

( >= 116 ) 47.73% 100.00% 99.94% 1.9e+04 0.5227

( >= 117 ) 45.45% 100.00% 99.94% 1.8e+04 0.5455

( >= 121 ) 43.18% 100.00% 99.93% 1.7e+04 0.5682

( >= 122 ) 40.91% 100.00% 99.93% 1.6e+04 0.5909

( >= 124 ) 38.64% 100.00% 99.93% 1.5e+04 0.6137

( >= 125 ) 36.36% 100.00% 99.93% 1.4e+04 0.6364

( >= 126 ) 36.36% 100.00% 99.93% 0.6364

( >= 129 ) 34.09% 100.00% 99.93% 0.6591

( >= 132 ) 31.82% 100.00% 99.92% 0.6818

( >= 137 ) 29.55% 100.00% 99.92% 0.7045

( >= 138 ) 27.27% 100.00% 99.92% 0.7273

( >= 143 ) 25.00% 100.00% 99.92% 0.7500

( >= 149 ) 22.73% 100.00% 99.91% 0.7727

( >= 155 ) 20.45% 100.00% 99.91% 0.7955

( >= 164 ) 18.18% 100.00% 99.91% 0.8182

( >= 166 ) 15.91% 100.00% 99.91% 0.8409

( >= 170 ) 13.64% 100.00% 99.90% 0.8636

( >= 171 ) 11.36% 100.00% 99.90% 0.8864

( >= 174 ) 9.09% 100.00% 99.90% 0.9091

( >= 176 ) 6.82% 100.00% 99.90% 0.9318

( >= 205 ) 4.55% 100.00% 99.89% 0.9545

( >= 210 ) 2.27% 100.00% 99.89% 0.9773

( > 210 ) 0.00% 100.00% 99.89% 1.0000

------------------------------------------------------------------------------

ROC Asymptotic normal

Obs area Std. err. [95% conf. interval]

------------------------------------------------------------

39,885 0.9997 0.0001 0.99953 0.99992

. cutpt EDOCase MEDREADS_EDO_Score , liu

Empirical cutpoint estimation

Method: Liu

Reference variable: EDOCase (0=neg, 1=pos)

Classification variable: MEDREADS_EDO_Score

Empirical optimal cutpoint: 44.5

Sensitivity at cutpoint: 1.00

Specificity at cutpoint: 1.00

Area under ROC curve at cutpoint: 1.00

. cutpt EDOCase MEDREADS_EDO_Score , youden

Empirical cutpoint estimation

Method: Youden

Reference variable: EDOCase (0=neg, 1=pos)

Classification variable: MEDREADS_EDO_Score

Empirical optimal cutpoint: 44.5

Youden index (J): 0.997

SE(J): 0.0003

Sensitivity at cutpoint: 1.00

Specificity at cutpoint: 1.00

Area under ROC curve at cutpoint: 1.00

. cutpt EDOCase MEDREADS_EDO_Score , nearest

Empirical cutpoint estimation

Method: Nearest to (0,1)

Reference variable: EDOCase (0=neg, 1=pos)

Classification variable: MEDREADS_EDO_Score

Empirical optimal cutpoint: 44.5

Sensitivity at cutpoint: 1.00

Specificity at cutpoint: 1.00

Area under ROC curve at cutpoint: 1.00

Sensitivity

. cii proportions 44 44

Binomial exact

Variable | Obs Proportion Std. err. [95% conf. interval]

-------------+---------------------------------------------------------------

| 44 1 0 .9195801 1*

(*) one-sided, 97.5% confidence interval

Specificity

. cii proportions 40008 39885

Binomial exact

Variable | Obs Proportion Std. err. [95% conf. interval]

-------------+---------------------------------------------------------------

| 40,008 .9969256 .0002768 .9963329 .9974443

Mania

. roctab ManiaCase MEDREADS_Mania_Score , graph summary title(Mania Cases) detail

Detailed report of sensitivity and specificity

------------------------------------------------------------------------------

Correctly

Cutpoint Sensitivity Specificity classified LR+ LR-

------------------------------------------------------------------------------

( >= -10 ) 100.00% 0.00% 0.56% 1.0000

( >= -8 ) 100.00% 0.01% 0.57% 1.0001 0.0000

( >= -7 ) 100.00% 0.04% 0.60% 1.0004 0.0000

( >= -6 ) 93.75% 71.03% 71.15% 3.2358 0.0880

( >= -5 ) 93.75% 71.03% 71.16% 3.2360 0.0880

( >= -4 ) 92.86% 71.22% 71.35% 3.2269 0.1003

( >= -3 ) 92.86% 71.23% 71.35% 3.2277 0.1003

( >= -2 ) 92.86% 71.51% 71.63% 3.2588 0.0999

( >= -1 ) 92.41% 71.65% 71.77% 3.2599 0.1059

( >= 0 ) 83.04% 94.08% 94.02% 14.0319 0.1803

( >= 1 ) 83.04% 94.15% 94.09% 14.1952 0.1802

( >= 2 ) 82.59% 94.70% 94.63% 15.5757 0.1839

( >= 3 ) 82.59% 94.72% 94.65% 15.6352 0.1838

( >= 4 ) 81.25% 95.14% 95.06% 16.7140 0.1971

( >= 5 ) 80.36% 95.66% 95.57% 18.4970 0.2053

( >= 6 ) 80.36% 95.67% 95.58% 18.5509 0.2053

( >= 7 ) 79.91% 95.72% 95.63% 18.6761 0.2099

( >= 8 ) 79.02% 97.23% 97.12% 28.4902 0.2158

( >= 9 ) 78.57% 97.39% 97.28% 30.0503 0.2200

( >= 10 ) 78.57% 97.42% 97.31% 30.4616 0.2200

( >= 11 ) 76.79% 97.98% 97.86% 37.9253 0.2369

( >= 12 ) 76.79% 98.02% 97.90% 38.7455 0.2368

( >= 13 ) 76.34% 98.04% 97.91% 38.8663 0.2413

( >= 14 ) 76.34% 98.04% 97.92% 39.0167 0.2413

( >= 15 ) 75.00% 98.27% 98.14% 43.3611 0.2544

( >= 16 ) 75.00% 98.27% 98.14% 43.4243 0.2544

( >= 17 ) 75.00% 98.33% 98.20% 44.8653 0.2543

( >= 18 ) 75.00% 98.35% 98.22% 45.4829 0.2542

( >= 20 ) 75.00% 98.37% 98.24% 45.9749 0.2541

( >= 21 ) 75.00% 98.42% 98.29% 47.5931 0.2540

( >= 22 ) 75.00% 98.44% 98.31% 48.1321 0.2540

( >= 23 ) 75.00% 98.45% 98.32% 48.2884 0.2539

( >= 24 ) 75.00% 98.45% 98.32% 48.3672 0.2539

( >= 26 ) 75.00% 98.47% 98.34% 48.9240 0.2539

( >= 27 ) 75.00% 98.48% 98.35% 49.4938 0.2538

( >= 28 ) 75.00% 98.49% 98.36% 49.5764 0.2538

( >= 29 ) 75.00% 98.50% 98.37% 49.9091 0.2538

( >= 30 ) 75.00% 98.50% 98.37% 49.9929 0.2538

( >= 32 ) 75.00% 98.53% 98.39% 50.8475 0.2537

( >= 33 ) 75.00% 98.56% 98.42% 51.9122 0.2537

( >= 34 ) 75.00% 98.58% 98.45% 52.7407 0.2536

( >= 35 ) 75.00% 98.62% 98.49% 54.4796 0.2535

( >= 36 ) 75.00% 98.63% 98.49% 54.5794 0.2535

( >= 37 ) 74.55% 98.65% 98.52% 55.2687 0.2579

( >= 38 ) 74.11% 98.66% 98.53% 55.4559 0.2624

( >= 39 ) 74.11% 98.67% 98.53% 55.6659 0.2624

( >= 40 ) 74.11% 98.70% 98.56% 56.8502 0.2623

( >= 41 ) 74.11% 98.72% 98.58% 57.8576 0.2623

( >= 42 ) 73.66% 98.87% 98.73% 65.0658 0.2664

( >= 43 ) 72.77% 98.89% 98.74% 65.2949 0.2754

( >= 44 ) 66.07% 98.90% 98.72% 60.2405 0.3430

( >= 45 ) 66.07% 98.94% 98.76% 62.5410 0.3429

( >= 46 ) 64.29% 98.98% 98.79% 63.1099 0.3608

( >= 47 ) 64.29% 98.99% 98.79% 63.5818 0.3608

( >= 48 ) 62.95% 99.07% 98.87% 68.0247 0.3740

( >= 49 ) 61.16% 99.08% 98.87% 66.8233 0.3920

( >= 50 ) 48.21% 99.17% 98.89% 58.2994 0.5222

( >= 51 ) 48.21% 99.19% 98.90% 59.3858 0.5221

( >= 52 ) 45.98% 99.23% 98.93% 59.4036 0.5444

( >= 53 ) 45.54% 99.23% 98.93% 59.4077 0.5488

( >= 54 ) 44.20% 99.25% 98.94% 59.0192 0.5622

( >= 55 ) 41.96% 99.27% 98.95% 57.3915 0.5846

( >= 56 ) 39.73% 99.40% 99.06% 65.9337 0.6063

( >= 57 ) 39.73% 99.41% 99.07% 67.0560 0.6063

( >= 58 ) 37.95% 99.43% 99.08% 66.5932 0.6241

( >= 59 ) 37.05% 99.43% 99.08% 65.3149 0.6331

( >= 60 ) 36.61% 99.47% 99.11% 68.8097 0.6373

( >= 61 ) 33.93% 99.59% 99.23% 83.5808 0.6634

( >= 62 ) 33.04% 99.60% 99.22% 81.8892 0.6724

( >= 63 ) 33.04% 99.61% 99.23% 83.9891 0.6723

( >= 64 ) 33.04% 99.64% 99.27% 92.2702 0.6720

( >= 65 ) 32.59% 99.66% 99.28% 95.7423 0.6764

( >= 66 ) 32.59% 99.66% 99.29% 97.1821 0.6764

( >= 67 ) 31.25% 99.76% 99.37% 129.1055 0.6892

( >= 68 ) 30.36% 99.76% 99.37% 128.0854 0.6981

( >= 70 ) 30.36% 99.77% 99.38% 130.8700 0.6980

( >= 71 ) 29.02% 99.81% 99.41% 151.4345 0.7112

( >= 72 ) 29.02% 99.81% 99.41% 153.4507 0.7112

( >= 74 ) 29.02% 99.82% 99.43% 164.4086 0.7111

( >= 76 ) 28.57% 99.82% 99.42% 161.8792 0.7155

( >= 77 ) 28.13% 99.84% 99.43% 171.6121 0.7199

( >= 79 ) 28.13% 99.84% 99.44% 174.2896 0.7199

( >= 80 ) 28.13% 99.85% 99.45% 185.9095 0.7198

( >= 81 ) 27.68% 99.86% 99.45% 192.5834 0.7243

( >= 82 ) 26.79% 99.86% 99.45% 186.3710 0.7332

( >= 84 ) 26.79% 99.87% 99.46% 200.4371 0.7331

( >= 86 ) 26.34% 99.87% 99.46% 200.8928 0.7376

( >= 87 ) 25.45% 99.87% 99.45% 194.0829 0.7465

( >= 88 ) 25.45% 99.88% 99.46% 205.9599 0.7465

( >= 89 ) 25.00% 99.88% 99.46% 206.5687 0.7509

( >= 90 ) 25.00% 99.89% 99.47% 220.3335 0.7509

( >= 92 ) 24.55% 99.90% 99.48% 243.4407 0.7552

( >= 93 ) 24.11% 99.91% 99.48% 265.5717 0.7596

( >= 94 ) 24.11% 99.92% 99.49% 289.7278 0.7596

( >= 95 ) 23.21% 99.92% 99.49% 278.9971 0.7685

( >= 96 ) 22.32% 99.92% 99.49% 285.5748 0.7774

( >= 98 ) 22.32% 99.93% 99.49% 305.2707 0.7774

( >= 99 ) 21.88% 99.93% 99.49% 321.3269 0.7818

( >= 100 ) 21.88% 99.93% 99.50% 333.7058 0.7818

( >= 101 ) 21.88% 99.94% 99.50% 361.5181 0.7817

( >= 102 ) 21.43% 99.95% 99.51% 404.7098 0.7861

( >= 103 ) 20.98% 99.95% 99.50% 396.2783 0.7906

( >= 104 ) 20.09% 99.97% 99.52% 612.9289 0.7994

( >= 105 ) 19.64% 99.97% 99.52% 599.3083 0.8038

( >= 106 ) 19.20% 99.97% 99.52% 634.4211 0.8083

( >= 107 ) 19.20% 99.97% 99.52% 761.3054 0.8082

( >= 108 ) 18.30% 99.98% 99.52% 1.0e+03 0.8171

( >= 109 ) 17.86% 99.98% 99.52% 1.0e+03 0.8216

( >= 111 ) 16.52% 99.98% 99.52% 1.1e+03 0.8349

( >= 112 ) 16.07% 99.99% 99.52% 1.3e+03 0.8394

( >= 113 ) 15.18% 99.99% 99.51% 1.5e+03 0.8483

( >= 114 ) 12.95% 99.99% 99.50% 1.3e+03 0.8706

( >= 115 ) 12.95% 99.99% 99.50% 1.7e+03 0.8706

( >= 116 ) 12.95% 99.99% 99.51% 2.6e+03 0.8706

( >= 117 ) 12.05% 99.99% 99.50% 2.4e+03 0.8795

( >= 118 ) 11.61% 99.99% 99.50% 2.3e+03 0.8840

( >= 122 ) 11.16% 99.99% 99.50% 2.2e+03 0.8884

( >= 124 ) 10.71% 99.99% 99.49% 2.1e+03 0.8929

( >= 129 ) 10.27% 99.99% 99.49% 2.0e+03 0.8974

( >= 130 ) 9.82% 100.00% 99.49% 3.9e+03 0.9018

( >= 132 ) 9.38% 100.00% 99.49% 3.7e+03 0.9063

( >= 141 ) 8.93% 100.00% 99.49% 3.5e+03 0.9107

( >= 142 ) 8.93% 100.00% 99.49% 0.9107

( >= 147 ) 8.48% 100.00% 99.49% 0.9152

( >= 155 ) 8.04% 100.00% 99.48% 0.9196

( >= 158 ) 7.59% 100.00% 99.48% 0.9241

( >= 159 ) 7.14% 100.00% 99.48% 0.9286

( >= 162 ) 6.70% 100.00% 99.48% 0.9330

( >= 166 ) 5.36% 100.00% 99.47% 0.9464

( >= 167 ) 4.91% 100.00% 99.47% 0.9509

( >= 169 ) 4.46% 100.00% 99.46% 0.9554

( >= 177 ) 4.02% 100.00% 99.46% 0.9598

( >= 193 ) 3.57% 100.00% 99.46% 0.9643

( >= 197 ) 3.13% 100.00% 99.46% 0.9688

( >= 202 ) 2.68% 100.00% 99.45% 0.9732

( >= 204 ) 2.23% 100.00% 99.45% 0.9777

( >= 222 ) 1.79% 100.00% 99.45% 0.9821

( >= 223 ) 1.34% 100.00% 99.45% 0.9866

( >= 273 ) 0.89% 100.00% 99.44% 0.9911

( >= 287 ) 0.45% 100.00% 99.44% 0.9955

( > 287 ) 0.00% 100.00% 99.44% 1.0000

------------------------------------------------------------------------------

ROC Asymptotic normal

Obs area Std. err. [95% conf. interval]

------------------------------------------------------------

39,885 0.9333 0.0107 0.91231 0.95427

. cutpt ManiaCase MEDREADS_Mania_Score , liu

Empirical cutpoint estimation

Method: Liu

Reference variable: ManiaCase (0=neg, 1=pos)

Classification variable: MEDREADS_Mania_Score

Empirical optimal cutpoint: 2.5

Sensitivity at cutpoint: 0.83

Specificity at cutpoint: 0.95

Area under ROC curve at cutpoint: 0.89

. cutpt ManiaCase MEDREADS_Mania_Score , youden

Empirical cutpoint estimation

Method: Youden

Reference variable: ManiaCase (0=neg, 1=pos)

Classification variable: MEDREADS_Mania_Score

Empirical optimal cutpoint: 2.5

Youden index (J): 0.773

SE(J): 0.0254

Sensitivity at cutpoint: 0.83

Specificity at cutpoint: 0.95

Area under ROC curve at cutpoint: 0.89

. cutpt ManiaCase MEDREADS_Mania_Score , nearest

Empirical cutpoint estimation

Method: Nearest to (0,1)

Reference variable: ManiaCase (0=neg, 1=pos)

Classification variable: MEDREADS_Mania_Score

Empirical optimal cutpoint: .5

Sensitivity at cutpoint: 0.83

Specificity at cutpoint: 0.94

Area under ROC curve at cutpoint: 0.89

. *Sensitivity

. cii proportions 224 185

Binomial exact

Variable | Obs Proportion Std. err. [95% conf. interval]

-------------+---------------------------------------------------------------

| 224 .8258929 .0253365 .7697673 .8731666

. *Specificity

. cii proportions 39661 37566

Binomial exact

Variable | Obs Proportion Std. err. [95% conf. interval]

-------------+---------------------------------------------------------------

| 39,661 .9471773 .0011232 .9449304 .9493585

> eet("Sheet1") firstrow

(153 vars, 39,885 obs)

. roctab PsychosisCase MEDREADS_Psychosis_Score , graph summary title(Psychosis Cases) detail

Detailed report of sensitivity and specificity

------------------------------------------------------------------------------

Correctly

Cutpoint Sensitivity Specificity classified LR+ LR-

------------------------------------------------------------------------------

( >= -11 ) 100.00% 0.00% 1.45% 1.0000

( >= -10 ) 100.00% 0.01% 1.46% 1.0001 0.0000

( >= -8 ) 100.00% 0.02% 1.47% 1.0002 0.0000

( >= -7 ) 100.00% 0.03% 1.48% 1.0003 0.0000

( >= -6 ) 100.00% 0.03% 1.49% 1.0003 0.0000

( >= -5 ) 100.00% 0.08% 1.53% 1.0008 0.0000

( >= -4 ) 100.00% 1.67% 3.10% 1.0170 0.0000

( >= -3 ) 100.00% 1.68% 3.11% 1.0171 0.0000

( >= -2 ) 100.00% 1.88% 3.31% 1.0192 0.0000

( >= -1 ) 100.00% 2.06% 3.49% 1.0210 0.0000

( >= 0 ) 100.00% 2.24% 3.66% 1.0229 0.0000

( >= 1 ) 100.00% 24.22% 25.33% 1.3197 0.0000

( >= 3 ) 100.00% 24.73% 25.83% 1.3286 0.0000

( >= 4 ) 100.00% 24.80% 25.89% 1.3297 0.0000

( >= 5 ) 100.00% 24.80% 25.90% 1.3298 0.0000

( >= 6 ) 100.00% 24.82% 25.91% 1.3302 0.0000

( >= 7 ) 100.00% 94.55% 94.63% 18.3411 0.0000

( >= 8 ) 100.00% 94.56% 94.64% 18.3754 0.0000

( >= 9 ) 100.00% 94.75% 94.83% 19.0524 0.0000

( >= 10 ) 100.00% 94.80% 94.87% 19.2200 0.0000

( >= 11 ) 100.00% 94.86% 94.94% 19.4676 0.0000

( >= 12 ) 100.00% 94.98% 95.05% 19.9113 0.0000

( >= 13 ) 100.00% 95.02% 95.09% 20.0843 0.0000

( >= 14 ) 100.00% 95.08% 95.15% 20.3126 0.0000

( >= 15 ) 100.00% 95.22% 95.29% 20.9180 0.0000

( >= 16 ) 100.00% 95.74% 95.80% 23.4797 0.0000

( >= 17 ) 100.00% 95.90% 95.96% 24.4131 0.0000

( >= 18 ) 100.00% 96.02% 96.08% 25.1311 0.0000

( >= 19 ) 100.00% 96.02% 96.08% 25.1472 0.0000

( >= 20 ) 100.00% 96.07% 96.13% 25.4402 0.0000

( >= 21 ) 100.00% 96.20% 96.25% 26.2910 0.0000

( >= 22 ) 100.00% 98.18% 98.20% 54.8954 0.0000

( >= 23 ) 100.00% 98.18% 98.21% 54.9720 0.0000

( >= 24 ) 100.00% 98.23% 98.25% 56.3918 0.0000

( >= 25 ) 100.00% 98.23% 98.26% 56.5538 0.0000

( >= 26 ) 100.00% 98.26% 98.29% 57.4635 0.0000

( >= 27 ) 100.00% 98.27% 98.29% 57.7166 0.0000

( >= 28 ) 100.00% 98.57% 98.59% 69.9379 0.0000

( >= 29 ) 100.00% 98.58% 98.60% 70.4389 0.0000

( >= 30 ) 100.00% 98.63% 98.65% 72.9223 0.0000

( >= 31 ) 100.00% 98.64% 98.66% 73.4671 0.0000

( >= 32 ) 100.00% 98.67% 98.69% 75.2971 0.0000

( >= 33 ) 100.00% 98.68% 98.70% 75.5868 0.0000

( >= 34 ) 100.00% 98.68% 98.70% 75.8782 0.0000

( >= 35 ) 100.00% 98.70% 98.72% 77.0685 0.0000

( >= 36 ) 100.00% 98.76% 98.77% 80.3782 0.0000

( >= 37 ) 100.00% 98.79% 98.81% 82.5734 0.0000

( >= 38 ) 100.00% 98.79% 98.81% 82.7475 0.0000

( >= 39 ) 99.66% 98.83% 98.84% 85.3363 0.0035

( >= 40 ) 99.48% 98.97% 98.98% 96.7868 0.0052

( >= 41 ) 99.31% 98.98% 98.98% 97.3416 0.0070

( >= 42 ) 98.79% 99.06% 99.05% 104.9480 0.0122

( >= 43 ) 98.79% 99.07% 99.07% 106.3853 0.0122

( >= 44 ) 96.55% 99.08% 99.04% 105.1238 0.0348

( >= 45 ) 96.03% 99.11% 99.07% 108.1552 0.0400

( >= 46 ) 96.03% 99.63% 99.58% 260.3183 0.0398

( >= 47 ) 90.86% 99.63% 99.51% 248.0107 0.0917

( >= 48 ) 89.31% 99.65% 99.50% 258.1162 0.1073

( >= 49 ) 88.10% 99.66% 99.49% 258.4289 0.1194

( >= 50 ) 85.17% 99.68% 99.47% 267.8178 0.1487

( >= 51 ) 83.62% 99.69% 99.46% 269.4015 0.1643

( >= 52 ) 83.62% 99.69% 99.46% 271.6315 0.1643

( >= 53 ) 79.83% 99.69% 99.41% 261.4679 0.2023

( >= 54 ) 79.48% 99.70% 99.41% 269.3163 0.2058

( >= 55 ) 78.62% 99.73% 99.42% 286.1300 0.2144

( >= 56 ) 76.38% 99.74% 99.40% 288.6649 0.2368

( >= 57 ) 75.69% 99.74% 99.39% 291.6680 0.2437

( >= 58 ) 75.34% 99.74% 99.39% 290.3392 0.2472

( >= 59 ) 74.83% 99.75% 99.39% 303.2059 0.2523

( >= 60 ) 73.62% 99.77% 99.39% 325.1326 0.2644

( >= 61 ) 72.76% 99.80% 99.41% 361.9897 0.2730

( >= 62 ) 72.59% 99.80% 99.41% 365.7691 0.2747

( >= 63 ) 72.24% 99.80% 99.40% 364.0314 0.2781

( >= 64 ) 72.07% 99.80% 99.40% 367.8866 0.2799

( >= 65 ) 71.21% 99.83% 99.42% 430.5790 0.2884

( >= 66 ) 70.69% 99.84% 99.42% 455.4829 0.2936

( >= 67 ) 70.34% 99.85% 99.42% 476.7198 0.2970

( >= 68 ) 69.83% 99.85% 99.42% 473.2145 0.3022

( >= 69 ) 69.31% 99.86% 99.41% 486.4863 0.3073

( >= 70 ) 68.79% 99.86% 99.41% 500.7136 0.3125

( >= 71 ) 67.93% 99.89% 99.43% 620.9538 0.3210

( >= 72 ) 67.93% 99.90% 99.43% 667.5055 0.3210

( >= 73 ) 67.76% 99.90% 99.43% 682.9110 0.3227

( >= 74 ) 67.41% 99.90% 99.43% 697.2902 0.3262

( >= 75 ) 66.90% 99.91% 99.43% 751.2222 0.3313

( >= 76 ) 66.38% 99.93% 99.44% 899.6452 0.3365

( >= 77 ) 64.14% 99.93% 99.41% 869.2676 0.3589

( >= 78 ) 63.97% 99.93% 99.41% 931.1515 0.3606

( >= 79 ) 63.45% 99.93% 99.40% 923.6219 0.3658

( >= 80 ) 63.45% 99.94% 99.41% 997.5161 0.3657

( >= 81 ) 61.90% 99.94% 99.38% 973.1204 0.3813

( >= 82 ) 61.72% 99.95% 99.40% 1.3e+03 0.3829

( >= 83 ) 60.69% 99.95% 99.38% 1.3e+03 0.3933

( >= 84 ) 60.69% 99.95% 99.38% 1.3e+03 0.3933

( >= 85 ) 60.17% 99.96% 99.38% 1.4e+03 0.3984

( >= 87 ) 59.48% 99.96% 99.37% 1.4e+03 0.4053

( >= 88 ) 58.62% 99.96% 99.36% 1.4e+03 0.4140

( >= 89 ) 57.76% 99.96% 99.35% 1.4e+03 0.4226

( >= 90 ) 57.24% 99.96% 99.34% 1.5e+03 0.4277

( >= 91 ) 55.86% 99.96% 99.32% 1.5e+03 0.4415

( >= 92 ) 55.52% 99.96% 99.32% 1.5e+03 0.4450

( >= 93 ) 54.48% 99.96% 99.30% 1.4e+03 0.4553

( >= 94 ) 53.97% 99.96% 99.29% 1.4e+03 0.4605

( >= 95 ) 53.28% 99.96% 99.28% 1.4e+03 0.4674

( >= 96 ) 53.28% 99.96% 99.29% 1.5e+03 0.4674

( >= 97 ) 52.93% 99.96% 99.28% 1.5e+03 0.4709

( >= 98 ) 52.76% 99.96% 99.28% 1.5e+03 0.4726

( >= 99 ) 52.41% 99.97% 99.28% 1.6e+03 0.4760

( >= 100 ) 51.03% 99.97% 99.26% 1.5e+03 0.4898

( >= 101 ) 50.34% 99.97% 99.25% 1.5e+03 0.4967

( >= 102 ) 49.48% 99.97% 99.23% 1.5e+03 0.5053

( >= 103 ) 49.31% 99.97% 99.23% 1.5e+03 0.5071

( >= 104 ) 49.14% 99.97% 99.23% 1.5e+03 0.5088

( >= 105 ) 48.97% 99.97% 99.23% 1.5e+03 0.5105

( >= 106 ) 48.45% 99.97% 99.22% 1.5e+03 0.5157

( >= 107 ) 47.93% 99.97% 99.21% 1.4e+03 0.5209

( >= 108 ) 46.55% 99.97% 99.20% 1.7e+03 0.5346

( >= 109 ) 46.21% 99.97% 99.19% 1.7e+03 0.5381

( >= 110 ) 46.03% 99.97% 99.19% 1.6e+03 0.5398

( >= 111 ) 45.52% 99.97% 99.18% 1.6e+03 0.5450

( >= 112 ) 44.83% 99.97% 99.17% 1.6e+03 0.5519

( >= 113 ) 44.66% 99.97% 99.17% 1.8e+03 0.5536

( >= 114 ) 43.45% 99.98% 99.16% 2.1e+03 0.5656

( >= 115 ) 42.93% 99.98% 99.15% 2.4e+03 0.5708

( >= 116 ) 42.59% 99.98% 99.15% 2.8e+03 0.5742

( >= 117 ) 42.07% 99.98% 99.14% 2.8e+03 0.5794

( >= 118 ) 41.55% 99.98% 99.14% 2.7e+03 0.5846

( >= 119 ) 40.17% 99.98% 99.11% 2.6e+03 0.5984

( >= 120 ) 39.31% 99.98% 99.10% 2.6e+03 0.6070

( >= 121 ) 38.10% 99.98% 99.08% 2.5e+03 0.6191

( >= 122 ) 37.76% 99.98% 99.08% 2.5e+03 0.6225

( >= 123 ) 37.59% 99.98% 99.08% 2.5e+03 0.6242

( >= 124 ) 37.41% 99.98% 99.07% 2.5e+03 0.6260

( >= 125 ) 37.07% 99.98% 99.07% 2.4e+03 0.6294

( >= 126 ) 36.72% 99.98% 99.06% 2.4e+03 0.6329

( >= 127 ) 36.21% 99.98% 99.06% 2.4e+03 0.6380

( >= 128 ) 35.52% 99.98% 99.05% 2.3e+03 0.6449

( >= 129 ) 35.00% 99.98% 99.04% 2.3e+03 0.6501

( >= 131 ) 34.66% 99.98% 99.03% 2.3e+03 0.6535

( >= 132 ) 34.31% 99.98% 99.03% 2.2e+03 0.6570

( >= 133 ) 34.14% 99.98% 99.03% 2.2e+03 0.6587

( >= 134 ) 33.97% 99.98% 99.02% 2.2e+03 0.6604

( >= 135 ) 33.62% 99.98% 99.02% 2.2e+03 0.6639

( >= 136 ) 33.28% 99.98% 99.01% 2.2e+03 0.6673

( >= 137 ) 32.93% 99.99% 99.01% 2.6e+03 0.6708

( >= 138 ) 32.76% 99.99% 99.01% 2.6e+03 0.6725

( >= 139 ) 32.24% 99.99% 99.00% 2.5e+03 0.6777

( >= 140 ) 31.90% 99.99% 99.00% 2.5e+03 0.6811

( >= 141 ) 31.55% 99.99% 98.99% 3.1e+03 0.6846

( >= 142 ) 31.03% 99.99% 98.99% 3.0e+03 0.6897

( >= 143 ) 30.52% 99.99% 98.98% 3.0e+03 0.6949

( >= 144 ) 30.00% 99.99% 98.97% 2.9e+03 0.7001

( >= 145 ) 29.66% 99.99% 98.97% 2.9e+03 0.7035

( >= 146 ) 29.31% 99.99% 98.96% 2.9e+03 0.7070

( >= 147 ) 28.97% 99.99% 98.96% 2.8e+03 0.7104

( >= 148 ) 28.79% 99.99% 98.95% 2.8e+03 0.7121

( >= 149 ) 28.10% 99.99% 98.94% 2.8e+03 0.7190

( >= 150 ) 27.93% 99.99% 98.94% 2.7e+03 0.7208

( >= 151 ) 27.59% 99.99% 98.94% 2.7e+03 0.7242

( >= 152 ) 27.41% 99.99% 98.93% 2.7e+03 0.7259

( >= 153 ) 27.24% 99.99% 98.93% 2.7e+03 0.7277

( >= 154 ) 26.55% 99.99% 98.92% 2.6e+03 0.7346

( >= 155 ) 26.03% 99.99% 98.91% 2.6e+03 0.7397

( >= 156 ) 25.86% 99.99% 98.91% 2.5e+03 0.7415

( >= 157 ) 25.17% 99.99% 98.90% 2.5e+03 0.7484

( >= 158 ) 24.66% 99.99% 98.89% 2.4e+03 0.7535

( >= 159 ) 24.66% 99.99% 98.90% 3.2e+03 0.7535

( >= 160 ) 23.97% 99.99% 98.89% 3.1e+03 0.7604

( >= 161 ) 23.62% 99.99% 98.88% 3.1e+03 0.7639

( >= 162 ) 23.45% 99.99% 98.88% 3.1e+03 0.7656

( >= 163 ) 22.41% 99.99% 98.87% 4.4e+03 0.7759

( >= 164 ) 22.24% 99.99% 98.86% 4.4e+03 0.7776

( >= 165 ) 22.07% 99.99% 98.86% 4.3e+03 0.7794

( >= 166 ) 21.90% 99.99% 98.86% 4.3e+03 0.7811

( >= 167 ) 21.38% 99.99% 98.85% 4.2e+03 0.7862

( >= 169 ) 20.52% 100.00% 98.84% 8.1e+03 0.7948

( >= 170 ) 20.17% 100.00% 98.84% 7.9e+03 0.7983

( >= 174 ) 19.83% 100.00% 98.83% 7.8e+03 0.8017

( >= 175 ) 19.14% 100.00% 98.82% 7.5e+03 0.8086

( >= 176 ) 18.97% 100.00% 98.82% 7.4e+03 0.8104

( >= 177 ) 18.79% 100.00% 98.82% 7.4e+03 0.8121

( >= 179 ) 18.45% 100.00% 98.81% 7.2e+03 0.8155

( >= 180 ) 18.28% 100.00% 98.81% 7.2e+03 0.8173

( >= 181 ) 18.10% 100.00% 98.81% 7.1e+03 0.8190

( >= 182 ) 17.59% 100.00% 98.80% 6.9e+03 0.8242

( >= 183 ) 17.41% 100.00% 98.80% 6.8e+03 0.8259

( >= 184 ) 17.07% 100.00% 98.79% 6.7e+03 0.8293

( >= 185 ) 16.90% 100.00% 98.79% 0.8310

( >= 186 ) 16.72% 100.00% 98.79% 0.8328

( >= 187 ) 16.55% 100.00% 98.79% 0.8345

( >= 189 ) 16.03% 100.00% 98.78% 0.8397

( >= 191 ) 15.69% 100.00% 98.77% 0.8431

( >= 193 ) 15.34% 100.00% 98.77% 0.8466

( >= 196 ) 15.17% 100.00% 98.77% 0.8483

( >= 197 ) 15.00% 100.00% 98.76% 0.8500

( >= 198 ) 14.31% 100.00% 98.75% 0.8569

( >= 201 ) 14.14% 100.00% 98.75% 0.8586

( >= 202 ) 13.97% 100.00% 98.75% 0.8603

( >= 203 ) 13.79% 100.00% 98.75% 0.8621

( >= 205 ) 13.45% 100.00% 98.74% 0.8655

( >= 206 ) 13.28% 100.00% 98.74% 0.8672

( >= 208 ) 12.93% 100.00% 98.73% 0.8707

( >= 211 ) 12.59% 100.00% 98.73% 0.8741

( >= 212 ) 12.24% 100.00% 98.72% 0.8776

( >= 213 ) 11.72% 100.00% 98.72% 0.8828

( >= 214 ) 11.38% 100.00% 98.71% 0.8862

( >= 215 ) 11.21% 100.00% 98.71% 0.8879

( >= 216 ) 11.03% 100.00% 98.71% 0.8897

( >= 217 ) 10.86% 100.00% 98.70% 0.8914

( >= 218 ) 10.69% 100.00% 98.70% 0.8931

( >= 219 ) 10.52% 100.00% 98.70% 0.8948

( >= 220 ) 10.34% 100.00% 98.70% 0.8966

( >= 224 ) 10.00% 100.00% 98.69% 0.9000

( >= 225 ) 9.66% 100.00% 98.69% 0.9034

( >= 226 ) 9.48% 100.00% 98.68% 0.9052

( >= 228 ) 9.31% 100.00% 98.68% 0.9069

( >= 229 ) 9.14% 100.00% 98.68% 0.9086

( >= 232 ) 8.79% 100.00% 98.67% 0.9121

( >= 233 ) 8.62% 100.00% 98.67% 0.9138

( >= 235 ) 8.28% 100.00% 98.67% 0.9172

( >= 236 ) 8.10% 100.00% 98.66% 0.9190

( >= 238 ) 7.59% 100.00% 98.66% 0.9241

( >= 240 ) 7.41% 100.00% 98.65% 0.9259

( >= 241 ) 7.07% 100.00% 98.65% 0.9293

( >= 249 ) 6.90% 100.00% 98.65% 0.9310

( >= 250 ) 6.21% 100.00% 98.64% 0.9379

( >= 251 ) 6.03% 100.00% 98.63% 0.9397

( >= 254 ) 5.86% 100.00% 98.63% 0.9414

( >= 256 ) 5.69% 100.00% 98.63% 0.9431

( >= 261 ) 5.52% 100.00% 98.63% 0.9448

( >= 264 ) 5.17% 100.00% 98.62% 0.9483

( >= 266 ) 5.00% 100.00% 98.62% 0.9500

( >= 269 ) 4.83% 100.00% 98.62% 0.9517

( >= 270 ) 4.48% 100.00% 98.61% 0.9552

( >= 274 ) 4.31% 100.00% 98.61% 0.9569

( >= 275 ) 3.97% 100.00% 98.60% 0.9603

( >= 278 ) 3.79% 100.00% 98.60% 0.9621

( >= 282 ) 3.62% 100.00% 98.60% 0.9638

( >= 283 ) 3.45% 100.00% 98.60% 0.9655

( >= 288 ) 3.28% 100.00% 98.59% 0.9672

( >= 291 ) 3.10% 100.00% 98.59% 0.9690

( >= 295 ) 2.41% 100.00% 98.58% 0.9759

( >= 298 ) 2.24% 100.00% 98.58% 0.9776

( >= 299 ) 1.90% 100.00% 98.57% 0.9810

( >= 300 ) 1.72% 100.00% 98.57% 0.9828

( >= 304 ) 1.55% 100.00% 98.57% 0.9845

( >= 306 ) 1.21% 100.00% 98.56% 0.9879

( >= 311 ) 1.03% 100.00% 98.56% 0.9897

( >= 319 ) 0.86% 100.00% 98.56% 0.9914

( >= 321 ) 0.69% 100.00% 98.56% 0.9931

( >= 324 ) 0.52% 100.00% 98.55% 0.9948

( >= 325 ) 0.00% 100.00% 98.55% 1.0000

( >= 326 ) 100.00% 0.00% 1.45% 1.0000

( >= 329 ) .% .% .%

( >= 336 ) .% .% .%

( >= 344 ) .% .% .%

( >= 368 ) .% .% .%

( >= 379 ) .% .% .%

( >= 404 ) .% .% .%

( > 404 ) .% .% .%

------------------------------------------------------------------------------

ROC Asymptotic normal

Obs area Std. err. [95% conf. interval]

------------------------------------------------------------

39,885 0.9987 0.0001 0.99841 0.99893

. cutpt PsychosisCase MEDREADS_Psychosis_Score , liu

Empirical cutpoint estimation

Method: Liu

Reference variable: PsychosisCase (0=neg, 1=pos)

Classification variable: MEDREADS_Psychosis_Score

Empirical optimal cutpoint: 37.5

Sensitivity at cutpoint: 1.00

Specificity at cutpoint: 0.99

Area under ROC curve at cutpoint: 0.99

. cutpt PsychosisCase MEDREADS_Psychosis_Score , youden

Empirical cutpoint estimation

Method: Youden

Reference variable: PsychosisCase (0=neg, 1=pos)

Classification variable: MEDREADS_Psychosis_Score

Empirical optimal cutpoint: 37.5

Youden index (J): 0.988

SE(J): 0.0006

Sensitivity at cutpoint: 1.00

Specificity at cutpoint: 0.99

Area under ROC curve at cutpoint: 0.99

. cutpt PsychosisCase MEDREADS_Psychosis_Score , nearest

Empirical cutpoint estimation

Method: Nearest to (0,1)

Reference variable: PsychosisCase (0=neg, 1=pos)

Classification variable: MEDREADS_Psychosis_Score

Empirical optimal cutpoint: 39.5

Sensitivity at cutpoint: 0.99

Specificity at cutpoint: 0.99

Area under ROC curve at cutpoint: 0.99

. *Sensitivity

. cii proportions 580 580

Binomial exact

Variable | Obs Proportion Std. err. [95% conf. interval]

-------------+---------------------------------------------------------------

| 580 1 0 .99366 1*

(*) one-sided, 97.5% confidence interval

. *Specificity

. cii proportions 39305 38830

Binomial exact

Variable | Obs Proportion Std. err. [95% conf. interval]

-------------+---------------------------------------------------------------

| 39,305 .987915 .0005511 .9867853 .9889718

.

. roctab Subtanceuse MEDREADS_Subst_Score , graph summary title(Psychosis Cases) detail

Detailed report of sensitivity and specificity

------------------------------------------------------------------------------

Correctly

Cutpoint Sensitivity Specificity classified LR+ LR-

------------------------------------------------------------------------------

( >= -12 ) 100.00% 0.00% 2.88% 1.0000

( >= -10 ) 97.73% 68.73% 69.56% 3.1250 0.0330

( >= -9 ) 97.38% 69.18% 69.99% 3.1601 0.0378

( >= -8 ) 97.30% 69.20% 70.01% 3.1591 0.0391

( >= -7 ) 97.30% 69.30% 70.10% 3.1692 0.0390

( >= -6 ) 97.30% 69.36% 70.16% 3.1753 0.0390

( >= -5 ) 97.30% 69.37% 70.17% 3.1761 0.0390

( >= -4 ) 97.04% 69.48% 70.27% 3.1796 0.0427

( >= -3 ) 96.95% 69.57% 70.35% 3.1857 0.0439

( >= -2 ) 96.34% 69.64% 70.40% 3.1729 0.0526

( >= -1 ) 96.34% 69.65% 70.42% 3.1745 0.0526

( >= 0 ) 95.73% 69.75% 70.49% 3.1641 0.0613

( >= 1 ) 95.64% 69.78% 70.52% 3.1644 0.0625

( >= 2 ) 95.55% 69.79% 70.53% 3.1632 0.0637

( >= 3 ) 95.55% 69.79% 70.54% 3.1635 0.0637

( >= 4 ) 94.07% 70.14% 70.83% 3.1502 0.0845

( >= 5 ) 93.72% 70.19% 70.87% 3.1442 0.0894

( >= 6 ) 93.55% 70.31% 70.97% 3.1504 0.0918

( >= 7 ) 88.49% 89.28% 89.26% 8.2562 0.1289

( >= 8 ) 88.49% 89.30% 89.28% 8.2702 0.1289

( >= 9 ) 87.18% 90.48% 90.38% 9.1576 0.1416

( >= 10 ) 87.10% 90.50% 90.40% 9.1659 0.1426

( >= 11 ) 86.92% 90.66% 90.55% 9.3017 0.1443

( >= 12 ) 86.57% 90.70% 90.58% 9.3106 0.1480

( >= 13 ) 85.00% 91.38% 91.19% 9.8560 0.1641

( >= 14 ) 84.22% 91.47% 91.26% 9.8744 0.1725

( >= 15 ) 84.13% 91.52% 91.31% 9.9182 0.1734

( >= 17 ) 84.13% 91.54% 91.32% 9.9394 0.1733

( >= 18 ) 83.96% 91.57% 91.35% 9.9552 0.1752

( >= 19 ) 83.87% 91.61% 91.39% 10.0000 0.1761

( >= 20 ) 83.61% 91.64% 91.41% 10.0058 0.1789

( >= 21 ) 83.61% 91.67% 91.44% 10.0337 0.1788

( >= 22 ) 80.21% 91.95% 91.62% 9.9684 0.2152

( >= 23 ) 79.60% 91.99% 91.63% 9.9340 0.2218

( >= 24 ) 79.60% 91.99% 91.63% 9.9372 0.2218

( >= 25 ) 79.25% 92.00% 91.64% 9.9096 0.2255

( >= 26 ) 79.25% 92.01% 91.64% 9.9128 0.2255

( >= 27 ) 79.08% 92.04% 91.67% 9.9359 0.2273

( >= 28 ) 78.99% 92.05% 91.67% 9.9314 0.2283

( >= 29 ) 78.73% 92.09% 91.71% 9.9534 0.2310

( >= 30 ) 78.55% 92.10% 91.71% 9.9444 0.2329

( >= 31 ) 78.38% 92.29% 91.89% 10.1648 0.2343

( >= 32 ) 78.29% 92.31% 91.90% 10.1773 0.2352

( >= 33 ) 78.29% 92.32% 91.92% 10.1979 0.2351

( >= 34 ) 77.94% 92.42% 92.01% 10.2873 0.2387

( >= 35 ) 77.16% 92.44% 92.00% 10.2012 0.2471

( >= 36 ) 76.72% 94.59% 94.07% 14.1729 0.2461

( >= 37 ) 76.37% 94.60% 94.07% 14.1354 0.2498

( >= 38 ) 76.02% 94.67% 94.13% 14.2617 0.2533

( >= 39 ) 75.68% 94.82% 94.27% 14.6138 0.2565

( >= 40 ) 75.24% 94.91% 94.35% 14.7876 0.2609

( >= 41 ) 74.54% 94.99% 94.41% 14.8923 0.2680

( >= 42 ) 74.02% 95.38% 94.76% 16.0098 0.2724

( >= 43 ) 73.50% 95.44% 94.81% 16.1217 0.2777

( >= 44 ) 72.97% 95.47% 94.83% 16.1256 0.2831

( >= 45 ) 72.71% 95.50% 94.84% 16.1508 0.2858

( >= 46 ) 72.10% 95.52% 94.85% 16.0890 0.2921

( >= 47 ) 71.58% 95.57% 94.88% 16.1491 0.2974

( >= 48 ) 71.14% 95.57% 94.87% 16.0600 0.3020

( >= 49 ) 70.88% 95.62% 94.91% 16.1706 0.3045

( >= 50 ) 70.44% 95.68% 94.96% 16.3113 0.3089

( >= 51 ) 70.10% 95.73% 94.99% 16.3972 0.3124

( >= 52 ) 68.79% 95.91% 95.13% 16.8121 0.3254

( >= 53 ) 68.18% 95.96% 95.16% 16.8759 0.3316

( >= 54 ) 66.87% 96.71% 95.85% 20.3170 0.3426

( >= 55 ) 65.48% 96.76% 95.86% 20.2102 0.3568

( >= 56 ) 65.13% 96.85% 95.94% 20.6793 0.3601

( >= 57 ) 63.82% 97.06% 96.11% 21.7241 0.3728

( >= 58 ) 61.64% 97.20% 96.18% 22.0478 0.3946

( >= 59 ) 60.77% 97.40% 96.34% 23.3532 0.4028

( >= 60 ) 59.02% 98.48% 97.34% 38.8193 0.4161

( >= 61 ) 58.24% 98.61% 97.45% 41.7788 0.4235

( >= 62 ) 57.11% 98.69% 97.50% 43.7183 0.4346

( >= 63 ) 56.50% 98.72% 97.51% 44.2123 0.4407

( >= 64 ) 56.06% 98.76% 97.53% 45.2423 0.4449

( >= 65 ) 55.01% 98.79% 97.53% 45.4393 0.4554

( >= 66 ) 54.66% 98.82% 97.55% 46.3368 0.4588

( >= 67 ) 53.88% 98.87% 97.57% 47.5442 0.4665

( >= 68 ) 53.44% 98.97% 97.66% 52.0174 0.4704

( >= 69 ) 53.10% 99.01% 97.69% 53.5625 0.4737

( >= 70 ) 52.66% 99.03% 97.70% 54.3976 0.4780

( >= 71 ) 52.48% 99.04% 97.70% 54.5083 0.4798

( >= 72 ) 52.48% 99.05% 97.71% 55.3990 0.4797

( >= 73 ) 51.79% 99.06% 97.70% 54.8124 0.4867

( >= 74 ) 51.61% 99.06% 97.70% 55.0793 0.4884

( >= 75 ) 51.00% 99.07% 97.69% 54.8816 0.4946

( >= 76 ) 50.57% 99.09% 97.69% 55.3351 0.4989

( >= 77 ) 50.04% 99.09% 97.68% 55.0735 0.5041

( >= 78 ) 50.04% 99.10% 97.69% 55.8672 0.5041

( >= 79 ) 49.96% 99.11% 97.70% 56.0928 0.5049

( >= 80 ) 49.26% 99.12% 97.69% 55.9588 0.5119

( >= 81 ) 48.30% 99.16% 97.70% 57.7482 0.5214

( >= 82 ) 47.95% 99.18% 97.71% 58.4129 0.5248

( >= 83 ) 47.69% 99.19% 97.71% 59.0225 0.5274

( >= 84 ) 47.60% 99.21% 97.72% 60.0659 0.5282

( >= 85 ) 47.25% 99.24% 97.74% 61.8418 0.5315

( >= 86 ) 47.17% 99.25% 97.75% 62.7878 0.5323

( >= 87 ) 46.82% 99.27% 97.76% 64.0854 0.5357

( >= 88 ) 46.56% 99.29% 97.77% 65.3440 0.5383

( >= 89 ) 46.29% 99.31% 97.79% 67.1670 0.5408

( >= 90 ) 45.60% 99.31% 97.77% 66.4043 0.5478

( >= 91 ) 45.25% 99.32% 97.77% 66.9021 0.5512

( >= 92 ) 45.16% 99.34% 97.78% 68.0725 0.5520

( >= 93 ) 44.73% 99.34% 97.77% 68.2116 0.5564

( >= 94 ) 44.38% 99.37% 97.79% 70.1658 0.5598

( >= 95 ) 44.20% 99.38% 97.79% 71.0497 0.5615

( >= 96 ) 43.59% 99.41% 97.81% 74.0643 0.5674

( >= 97 ) 43.16% 99.41% 97.80% 73.6473 0.5718

( >= 98 ) 42.20% 99.42% 97.78% 72.9750 0.5814

( >= 99 ) 41.85% 99.44% 97.78% 74.3627 0.5848

( >= 100 ) 41.06% 99.45% 97.77% 74.6815 0.5926

( >= 101 ) 40.54% 99.48% 97.79% 78.1327 0.5977

( >= 102 ) 39.84% 99.51% 97.80% 82.0976 0.6045

( >= 103 ) 39.49% 99.53% 97.80% 83.6028 0.6079

( >= 104 ) 38.71% 99.55% 97.80% 85.2002 0.6157

( >= 105 ) 37.93% 99.59% 97.82% 93.5753 0.6233

( >= 106 ) 37.14% 99.63% 97.83% 99.2227 0.6310

( >= 107 ) 36.18% 99.67% 97.84% 109.5006 0.6403

( >= 108 ) 35.05% 99.69% 97.83% 113.1423 0.6515

( >= 109 ) 34.61% 99.70% 97.83% 116.5909 0.6558

( >= 110 ) 33.83% 99.72% 97.82% 120.2229 0.6636

( >= 111 ) 33.13% 99.74% 97.82% 127.0674 0.6704

( >= 112 ) 32.52% 99.78% 97.85% 148.2062 0.6763

( >= 113 ) 31.91% 99.80% 97.84% 156.4691 0.6823

( >= 114 ) 31.21% 99.85% 97.88% 212.1228 0.6889

( >= 115 ) 30.34% 99.87% 97.87% 226.0146 0.6975

( >= 116 ) 29.73% 99.87% 97.86% 235.0263 0.7036

( >= 117 ) 29.29% 99.88% 97.85% 236.4139 0.7079

( >= 118 ) 28.77% 99.88% 97.83% 237.1421 0.7132

( >= 119 ) 27.90% 99.88% 97.81% 240.1655 0.7218

( >= 120 ) 27.38% 99.90% 97.81% 271.9154 0.7270

( >= 121 ) 26.94% 99.90% 97.80% 274.6196 0.7313

( >= 122 ) 26.68% 99.91% 97.80% 295.2860 0.7339

( >= 123 ) 26.42% 99.91% 97.80% 300.9818 0.7365

( >= 124 ) 26.07% 99.91% 97.79% 297.0085 0.7400

( >= 125 ) 25.89% 99.92% 97.79% 323.5705 0.7417

( >= 126 ) 25.54% 99.92% 97.78% 319.2126 0.7451

( >= 127 ) 25.46% 99.92% 97.78% 318.1232 0.7460

( >= 128 ) 25.02% 99.92% 97.77% 312.6759 0.7504

( >= 129 ) 24.50% 99.92% 97.75% 316.3326 0.7556

( >= 130 ) 24.24% 99.93% 97.75% 323.7679 0.7582

( >= 131 ) 23.54% 99.93% 97.73% 314.4508 0.7652

( >= 132 ) 22.84% 99.93% 97.71% 316.0199 0.7721

( >= 133 ) 22.67% 99.93% 97.71% 313.6075 0.7739

( >= 134 ) 22.41% 99.93% 97.70% 309.9890 0.7765

( >= 135 ) 22.06% 99.93% 97.69% 328.6491 0.7799

( >= 136 ) 21.71% 99.93% 97.68% 323.4531 0.7834

( >= 137 ) 21.53% 99.93% 97.68% 320.8550 0.7852

( >= 138 ) 21.36% 99.94% 97.68% 330.9738 0.7869

( >= 139 ) 21.10% 99.94% 97.67% 340.5696 0.7895

( >= 140 ) 20.84% 99.94% 97.66% 336.3476 0.7921

( >= 141 ) 20.58% 99.94% 97.66% 332.1257 0.7947

( >= 142 ) 20.23% 99.95% 97.66% 391.7765 0.7981

( >= 143 ) 19.97% 99.96% 97.66% 454.9495 0.8007

( >= 144 ) 19.88% 99.96% 97.65% 452.9627 0.8016

( >= 145 ) 19.70% 99.96% 97.65% 448.9894 0.8033

( >= 146 ) 19.62% 99.96% 97.65% 447.0027 0.8042

( >= 147 ) 19.18% 99.96% 97.64% 464.3559 0.8085

( >= 148 ) 18.83% 99.96% 97.63% 486.3671 0.8120

( >= 150 ) 18.13% 99.96% 97.61% 468.3535 0.8190

( >= 151 ) 18.05% 99.96% 97.61% 466.1018 0.8198

( >= 152 ) 17.79% 99.96% 97.60% 492.1225 0.8224

( >= 153 ) 17.52% 99.97% 97.60% 522.2607 0.8250

( >= 154 ) 17.26% 99.97% 97.59% 514.4657 0.8277

( >= 156 ) 16.83% 99.97% 97.58% 501.4742 0.8320

( >= 157 ) 16.65% 99.97% 97.57% 496.2776 0.8338

( >= 158 ) 16.13% 99.97% 97.56% 567.9915 0.8389

( >= 159 ) 15.87% 99.97% 97.56% 614.5929 0.8415

( >= 160 ) 15.61% 99.98% 97.55% 671.7571 0.8441

( >= 161 ) 14.91% 99.98% 97.53% 641.7344 0.8511

( >= 162 ) 14.56% 99.98% 97.52% 626.7230 0.8546

( >= 163 ) 13.86% 99.98% 97.50% 596.7004 0.8616

( >= 164 ) 13.51% 99.98% 97.49% 654.3196 0.8650

( >= 165 ) 12.99% 99.98% 97.48% 628.9911 0.8703

( >= 166 ) 12.47% 99.98% 97.46% 603.6626 0.8755

( >= 167 ) 12.12% 99.98% 97.45% 586.7770 0.8790

( >= 168 ) 11.77% 99.98% 97.44% 569.8913 0.8825

( >= 169 ) 11.25% 99.98% 97.43% 544.5628 0.8877

( >= 170 ) 10.99% 99.98% 97.43% 709.2854 0.8903

( >= 171 ) 10.72% 99.98% 97.42% 692.3977 0.8929

( >= 172 ) 10.55% 99.98% 97.41% 681.1392 0.8946

( >= 173 ) 10.37% 99.99% 97.41% 1.0e+03 0.8963

( >= 174 ) 10.03% 99.99% 97.40% 971.2847 0.8998

( >= 175 ) 9.76% 99.99% 97.40% 945.9469 0.9024

( >= 176 ) 9.68% 99.99% 97.39% 937.5008 0.9033

( >= 178 ) 9.33% 99.99% 97.39% 1.8e+03 0.9068

( >= 179 ) 9.15% 99.99% 97.38% 1.8e+03 0.9085

( >= 180 ) 9.07% 99.99% 97.38% 1.8e+03 0.9094

( >= 181 ) 8.98% 99.99% 97.38% 1.7e+03 0.9102

( >= 182 ) 8.89% 100.00% 97.38% 3.4e+03 0.9111

( >= 183 ) 8.72% 100.00% 97.37% 3.4e+03 0.9128

( >= 184 ) 8.54% 100.00% 97.37% 3.3e+03 0.9146

( >= 185 ) 8.20% 100.00% 97.36% 3.2e+03 0.9181

( >= 186 ) 7.93% 100.00% 97.35% 3.1e+03 0.9207

( >= 187 ) 7.85% 100.00% 97.35% 3.0e+03 0.9216

( >= 188 ) 7.76% 100.00% 97.34% 3.0e+03 0.9224

( >= 189 ) 7.67% 100.00% 97.34% 3.0e+03 0.9233

( >= 190 ) 7.32% 100.00% 97.33% 2.8e+03 0.9268

( >= 191 ) 7.06% 100.00% 97.32% 2.7e+03 0.9294

( >= 192 ) 6.80% 100.00% 97.32% 2.6e+03 0.9320

( >= 193 ) 6.63% 100.00% 97.31% 2.6e+03 0.9338

( >= 194 ) 6.54% 100.00% 97.31% 2.5e+03 0.9346

( >= 195 ) 6.36% 100.00% 97.30% 2.5e+03 0.9364

( >= 197 ) 6.28% 100.00% 97.30% 2.4e+03 0.9373

( >= 198 ) 6.10% 100.00% 97.30% 2.4e+03 0.9390

( >= 199 ) 5.93% 100.00% 97.29% 2.3e+03 0.9407

( >= 200 ) 5.84% 100.00% 97.29% 2.3e+03 0.9416

( >= 201 ) 5.75% 100.00% 97.29% 2.2e+03 0.9425

( >= 202 ) 5.67% 100.00% 97.28% 2.2e+03 0.9434

( >= 203 ) 5.58% 100.00% 97.28% 2.2e+03 0.9442

( >= 204 ) 5.49% 100.00% 97.28% 2.1e+03 0.9451

( >= 206 ) 5.41% 100.00% 97.28% 2.1e+03 0.9460

( >= 209 ) 5.14% 100.00% 97.27% 2.0e+03 0.9486

( >= 210 ) 4.80% 100.00% 97.26% 1.9e+03 0.9521

( >= 212 ) 4.62% 100.00% 97.25% 1.8e+03 0.9538

( >= 213 ) 4.27% 100.00% 97.24% 1.7e+03 0.9573

( >= 214 ) 4.18% 100.00% 97.24% 1.6e+03 0.9582

( >= 215 ) 4.01% 100.00% 97.24% 1.6e+03 0.9599

( >= 217 ) 3.75% 100.00% 97.23% 1.5e+03 0.9625

( >= 218 ) 3.66% 100.00% 97.23% 0.9634

( >= 219 ) 3.57% 100.00% 97.23% 0.9643

( >= 220 ) 3.40% 100.00% 97.22% 0.9660

( >= 221 ) 3.31% 100.00% 97.22% 0.9669

( >= 222 ) 2.96% 100.00% 97.21% 0.9704

( >= 224 ) 2.88% 100.00% 97.21% 0.9712

( >= 225 ) 2.79% 100.00% 97.20% 0.9721

( >= 230 ) 2.62% 100.00% 97.20% 0.9738

( >= 234 ) 2.53% 100.00% 97.20% 0.9747

( >= 237 ) 2.27% 100.00% 97.19% 0.9773

( >= 238 ) 2.18% 100.00% 97.19% 0.9782

( >= 243 ) 2.09% 100.00% 97.18% 0.9791

( >= 247 ) 1.92% 100.00% 97.18% 0.9808

( >= 248 ) 1.83% 100.00% 97.18% 0.9817

( >= 254 ) 1.74% 100.00% 97.17% 0.9826

( >= 255 ) 1.57% 100.00% 97.17% 0.9843

( >= 256 ) 1.48% 100.00% 97.17% 0.9852

( >= 257 ) 1.39% 100.00% 97.16% 0.9861

( >= 258 ) 1.31% 100.00% 97.16% 0.9869

( >= 262 ) 1.22% 100.00% 97.16% 0.9878

( >= 263 ) 1.13% 100.00% 97.16% 0.9887

( >= 266 ) 1.05% 100.00% 97.15% 0.9895

( >= 275 ) 0.96% 100.00% 97.15% 0.9904

( >= 276 ) 0.78% 100.00% 97.15% 0.9922

( >= 287 ) 0.70% 100.00% 97.14% 0.9930

( >= 291 ) 0.61% 100.00% 97.14% 0.9939

( >= 308 ) 0.52% 100.00% 97.14% 0.9948

( >= 325 ) 0.44% 100.00% 97.14% 0.9956

( >= 330 ) 0.35% 100.00% 97.13% 0.9965

( >= 340 ) 0.26% 100.00% 97.13% 0.9974

( >= 357 ) 0.17% 100.00% 97.13% 0.9983

( >= 361 ) 0.09% 100.00% 97.13% 0.9991

( > 361 ) 0.00% 100.00% 97.12% 1.0000

------------------------------------------------------------------------------

ROC Asymptotic normal

Obs area Std. err. [95% conf. interval]

------------------------------------------------------------

39,885 0.9436 0.0035 0.93675 0.95039

. cutpt Subtanceuse MEDREADS_Subst_Score , liu

Empirical cutpoint estimation

Method: Liu

Reference variable: Subtanceuse (0=neg, 1=pos)

Classification variable: MEDREADS_Subst_Score

Empirical optimal cutpoint: 7.5

Sensitivity at cutpoint: 0.88

Specificity at cutpoint: 0.89

Area under ROC curve at cutpoint: 0.89

. cutpt Subtanceuse MEDREADS_Subst_Score , youden

Empirical cutpoint estimation

Method: Youden

Reference variable: Subtanceuse (0=neg, 1=pos)

Classification variable: MEDREADS_Subst_Score

Empirical optimal cutpoint: 7.5

Youden index (J): 0.778

SE(J): 0.0096

Sensitivity at cutpoint: 0.88

Specificity at cutpoint: 0.89

Area under ROC curve at cutpoint: 0.89

. cutpt Subtanceuse MEDREADS_Subst_Score , nearest

Empirical cutpoint estimation

Method: Nearest to (0,1)

Reference variable: Subtanceuse (0=neg, 1=pos)

Classification variable: MEDREADS_Subst_Score

Empirical optimal cutpoint: 7.5

Sensitivity at cutpoint: 0.88

Specificity at cutpoint: 0.89

Area under ROC curve at cutpoint: 0.89

. *Sensitivity

. cii prop 1147 1015

Binomial exact

Variable | Obs Proportion Std. err. [95% conf. interval]

-------------+---------------------------------------------------------------

| 1,147 .8849172 .0094227 .8650185 .9028158

. display 4145 + 34593

38738

. *Specificity

. cii prop 38738 34593

Binomial exact

Variable | Obs Proportion Std. err. [95% conf. interval]

-------------+---------------------------------------------------------------

| 38,738 .8929991 .0015705 .8898785 .8960607

> eet("Sheet1") firstrow

(147 vars, 39,885 obs)

. roctab RaterRatedSuicidalPresentati MEDREADS_Suicidal_Score , graph summary title(Suicidal Cases) detail

Detailed report of sensitivity and specificity

------------------------------------------------------------------------------

Correctly

Cutpoint Sensitivity Specificity classified LR+ LR-

------------------------------------------------------------------------------

( >= -9 ) 100.00% 0.00% 2.92% 1.0000

( >= -7 ) 100.00% 0.00% 2.93% 1.0000 0.0000

( >= -6 ) 100.00% 0.01% 2.94% 1.0001 0.0000

( >= -5 ) 100.00% 0.04% 2.96% 1.0004 0.0000

( >= -4 ) 100.00% 0.06% 2.98% 1.0006 0.0000

( >= -3 ) 99.91% 0.17% 3.08% 1.0008 0.5108

( >= -2 ) 99.83% 0.56% 3.47% 1.0039 0.3046

( >= -1 ) 99.74% 2.51% 5.36% 1.0231 0.1024

( >= 0 ) 99.57% 14.39% 16.88% 1.1631 0.0298

( >= 1 ) 98.54% 76.70% 77.34% 4.2288 0.0190

( >= 2 ) 97.86% 94.78% 94.87% 18.7559 0.0226

( >= 3 ) 97.34% 95.61% 95.66% 22.1953 0.0278

( >= 4 ) 96.66% 96.01% 96.03% 24.2528 0.0348

( >= 5 ) 95.97% 96.92% 96.89% 31.1192 0.0416

( >= 6 ) 95.03% 97.59% 97.51% 39.3487 0.0510

( >= 7 ) 93.57% 98.18% 98.04% 51.3123 0.0655

( >= 8 ) 91.85% 98.83% 98.62% 78.3313 0.0824

( >= 9 ) 90.48% 99.17% 98.91% 108.7931 0.0960

( >= 10 ) 88.77% 99.31% 99.00% 128.2361 0.1131

( >= 11 ) 87.31% 99.40% 99.04% 144.4559 0.1277

( >= 12 ) 85.08% 99.47% 99.05% 159.8996 0.1500

( >= 13 ) 82.50% 99.53% 99.03% 174.5545 0.1758

( >= 14 ) 80.53% 99.59% 99.03% 197.3388 0.1955

( >= 15 ) 77.53% 99.66% 99.01% 227.4043 0.2255

( >= 16 ) 73.76% 99.74% 98.98% 285.5616 0.2631

( >= 17 ) 71.44% 99.78% 98.95% 317.9266 0.2862

( >= 18 ) 68.61% 99.81% 98.90% 363.8948 0.3145

( >= 19 ) 65.95% 99.86% 98.86% 455.9794 0.3410

( >= 20 ) 62.01% 99.88% 98.77% 500.1454 0.3804

( >= 21 ) 57.98% 99.90% 98.68% 590.6745 0.4207

( >= 22 ) 54.97% 99.92% 98.61% 686.5734 0.4506

( >= 23 ) 51.54% 99.93% 98.51% 688.1178 0.4849

( >= 24 ) 48.54% 99.93% 98.43% 671.2206 0.5150

( >= 25 ) 46.91% 99.94% 98.39% 726.5648 0.5312

( >= 26 ) 45.11% 99.95% 98.34% 831.7419 0.5492

( >= 27 ) 43.05% 99.95% 98.29% 926.0035 0.5697

( >= 28 ) 39.88% 99.96% 98.20% 965.1296 0.6014

( >= 29 ) 37.31% 99.96% 98.13% 962.9593 0.6272

( >= 30 ) 35.08% 99.97% 98.07% 1.0e+03 0.6494

( >= 31 ) 32.85% 99.97% 98.01% 1.2e+03 0.6717

( >= 32 ) 30.96% 99.97% 97.96% 1.2e+03 0.6906

( >= 33 ) 28.39% 99.98% 97.89% 1.8e+03 0.7162

( >= 34 ) 26.24% 99.99% 97.83% 2.0e+03 0.7377

( >= 35 ) 24.01% 99.99% 97.77% 2.3e+03 0.7599

( >= 36 ) 22.56% 99.99% 97.73% 2.9e+03 0.7745

( >= 37 ) 21.27% 99.99% 97.69% 2.7e+03 0.7874

( >= 38 ) 18.87% 99.99% 97.62% 2.4e+03 0.8114

( >= 39 ) 17.58% 99.99% 97.59% 3.4e+03 0.8242

( >= 40 ) 15.61% 99.99% 97.53% 3.0e+03 0.8440

( >= 41 ) 14.41% 99.99% 97.49% 2.8e+03 0.8560

( >= 42 ) 13.21% 99.99% 97.46% 2.6e+03 0.8680

( >= 43 ) 11.75% 99.99% 97.41% 2.3e+03 0.8825

( >= 44 ) 11.15% 99.99% 97.40% 2.2e+03 0.8886

( >= 45 ) 10.21% 99.99% 97.37% 2.0e+03 0.8980

( >= 46 ) 9.43% 99.99% 97.35% 1.8e+03 0.9057

( >= 47 ) 8.23% 99.99% 97.31% 1.6e+03 0.9177

( >= 48 ) 7.12% 99.99% 97.28% 1.4e+03 0.9289

( >= 49 ) 6.78% 99.99% 97.27% 1.3e+03 0.9323

( >= 50 ) 6.00% 99.99% 97.25% 1.2e+03 0.9400

( >= 51 ) 5.23% 99.99% 97.22% 1.0e+03 0.9477

( >= 52 ) 4.63% 99.99% 97.21% 896.6366 0.9537

( >= 53 ) 4.20% 99.99% 97.19% 813.6147 0.9580

( >= 54 ) 3.60% 99.99% 97.18% 697.3840 0.9640

( >= 55 ) 3.00% 100.00% 97.16% 1.2e+03 0.9700

( >= 56 ) 2.66% 100.00% 97.15% 1.0e+03 0.9734

( >= 57 ) 2.40% 100.00% 97.14% 931.2209 0.9760

( >= 58 ) 2.14% 100.00% 97.14% 831.4472 0.9786

( >= 59 ) 1.89% 100.00% 97.13% 731.6735 0.9812

( >= 60 ) 1.80% 100.00% 97.13% 698.4157 0.9820

( >= 61 ) 1.72% 100.00% 97.12% 665.1577 0.9829

( >= 62 ) 1.63% 100.00% 97.12% 0.9837

( >= 63 ) 1.20% 100.00% 97.11% 0.9880

( >= 65 ) 0.94% 100.00% 97.10% 0.9906

( >= 66 ) 0.86% 100.00% 97.10% 0.9914

( >= 67 ) 0.77% 100.00% 97.10% 0.9923

( >= 68 ) 0.69% 100.00% 97.10% 0.9931

( >= 69 ) 0.60% 100.00% 97.09% 0.9940

( >= 71 ) 0.34% 100.00% 97.09% 0.9966

( >= 72 ) 0.26% 100.00% 97.08% 0.9974

( >= 73 ) 0.17% 100.00% 97.08% 0.9983

( >= 77 ) 0.09% 100.00% 97.08% 0.9991

( >= 79 ) 0.00% 100.00% 97.08% 1.0000

( >= 80 ) 100.00% 0.00% 2.92% 1.0000

( > 80 ) .% .% .%

------------------------------------------------------------------------------

ROC Asymptotic normal

Obs area Std. err. [95% conf. interval]

------------------------------------------------------------

39,883 0.9863 0.0025 0.98150 0.99115

. cutpt RaterRatedSuicidalPresentati MEDREADS_Suicidal_Score , liu

Empirical cutpoint estimation

Method: Liu

Reference variable: RaterRatedSuicidalPresentati (0=neg, 1=pos)

Classification variable: MEDREADS_Suicidal_Score

Empirical optimal cutpoint: 2.5

Sensitivity at cutpoint: 0.97

Specificity at cutpoint: 0.96

Area under ROC curve at cutpoint: 0.96

. cutpt RaterRatedSuicidalPresentati MEDREADS_Suicidal_Score , youden

Empirical cutpoint estimation

Method: Youden

Reference variable: RaterRatedSuicidalPresentati (0=neg, 1=pos)

Classification variable: MEDREADS_Suicidal_Score

Empirical optimal cutpoint: 2.5

Youden index (J): 0.930

SE(J): 0.0048

Sensitivity at cutpoint: 0.97

Specificity at cutpoint: 0.96

Area under ROC curve at cutpoint: 0.96

. cutpt RaterRatedSuicidalPresentati MEDREADS_Suicidal_Score , nearest

Empirical cutpoint estimation

Method: Nearest to (0,1)

Reference variable: RaterRatedSuicidalPresentati (0=neg, 1=pos)

Classification variable: MEDREADS_Suicidal_Score

Empirical optimal cutpoint: 4.5

Sensitivity at cutpoint: 0.96

Specificity at cutpoint: 0.97

Area under ROC curve at cutpoint: 0.96

. display 1135+31

1166

Sensitivity

. cii prop 1166 1135

Binomial exact

Variable | Obs Proportion Std. err. [95% conf. interval]

-------------+---------------------------------------------------------------

| 1,166 .9734134 .0047112 .9624734 .9818658

. display 1698+37019

38717

Specificity

. cii prop 38717 37019

Binomial exact

Variable | Obs Proportion Std. err. [95% conf. interval]

-------------+---------------------------------------------------------------

| 38,717 .9561433 .0010407 .9540561 .9581617

.

28/4/2022

MedReads Prevalence estimates for each month 2019-2021

Eg 1 2019_10 is the prevalence estimate for October 2019.

firstrow

(145 vars, 128,501 obs)

Suicidal Cases

. encode Year_Month, gen(YearMonthCat)

. proportion MEDREADS_Suicidal_Case, over(YearMonthCat)

Proportion estimation Number of obs = 128,501

-------------------------------------------------------------------------------------

| Logit

| Proportion Std. err. [95% conf. interval]

------------------------------------+------------------------------------------------

MEDREADS_Suicidal_Case@YearMonthCat |

0 2019_10 | .9190979 .0022079 .9146635 .9233212

0 2019_11 | .9100297 .0022988 .9054213 .9144347

0 2019_12 | .9178667 .0021917 .9134674 .9220614

0 2019_4 | .9246728 .0029328 .9187193 .9302233

0 2019_5 | .928601 .0021397 .9242919 .9326827

0 2019_6 | .9289966 .002162 .9246406 .933119

0 2019_7 | .9324182 .0020393 .9283098 .9363072

0 2019_8 | .9288481 .0020657 .9246915 .932792

0 2019_9 | .922868 .0021984 .9184473 .9270681

1 2019_10 | .0809021 .0022079 .0766788 .0853365

1 2019_11 | .0899703 .0022988 .0855653 .0945787

1 2019_12 | .0821333 .0021917 .0779386 .0865326

1 2019_4 | .0753272 .0029328 .0697767 .0812807

1 2019_5 | .071399 .0021397 .0673173 .0757081

1 2019_6 | .0710034 .002162 .066881 .0753594

1 2019_7 | .0675818 .0020393 .0636928 .0716902

1 2019_8 | .0711519 .0020657 .067208 .0753085

1 2019_9 | .077132 .0021984 .0729319 .0815527

-------------------------------------------------------------------------------------

EDO

. proportion MEDREADS_EDO_Case , over(YearMonthCat)

Proportion estimation Number of obs = 128,501

--------------------------------------------------------------------------------

| Logit

| Proportion Std. err. [95% conf. interval]

-------------------------------+------------------------------------------------

MEDREADS_EDO_Case@YearMonthCat |

0 2019_10 | .9965253 .0004765 .9954545 .9973445

0 2019_11 | .9967084 .0004602 .9956714 .9974976

0 2019_12 | .9968141 .0004498 .9957989 .9975845

0 2019_4 | .9976537 .0005376 .9963246 .998503

0 2019_5 | .9975832 .000408 .9966358 .9982643

0 2019_6 | .9965986 .0004901 .9954893 .9974358

0 2019_7 | .9965021 .0004796 .9954242 .9973268

0 2019_8 | .9968363 .0004512 .9958164 .9976081

0 2019_9 | .9961298 .0005116 .9949859 .9970136

1 2019_10 | .0034747 .0004765 .0026555 .0045455

1 2019_11 | .0032916 .0004602 .0025024 .0043286

1 2019_12 | .0031859 .0004498 .0024155 .0042011

1 2019_4 | .0023463 .0005376 .001497 .0036754

1 2019_5 | .0024168 .000408 .0017357 .0033642

1 2019_6 | .0034014 .0004901 .0025642 .0045107

1 2019_7 | .0034979 .0004796 .0026732 .0045758

1 2019_8 | .0031637 .0004512 .0023919 .0041836

1 2019_9 | .0038702 .0005116 .0029864 .0050141

--------------------------------------------------------------------------------

Mania

. proportion MEDREADS_Mania_Case , over(YearMonthCat)

Proportion estimation Number of obs = 128,501

----------------------------------------------------------------------------------

| Logit

| Proportion Std. err. [95% conf. interval]

---------------------------------+------------------------------------------------

MEDREADS_Mania_Case@YearMonthCat |

0 2019_10 | .9814463 .0010926 .979179 .9834708

0 2019_11 | .980444 .0011124 .9781403 .9825093

0 2019_12 | .9808207 .0010948 .9785529 .9828529

0 2019_4 | .9813534 .0015032 .9781671 .9840823

0 2019_5 | .9813562 .001124 .9790206 .9834361

0 2019_6 | .9815051 .0011342 .979146 .9836018

0 2019_7 | .9827086 .001059 .9805058 .9846663

0 2019_8 | .9806302 .0011074 .9783361 .9826856

0 2019_9 | .981396 .0011134 .9790834 .9834572

1 2019_10 | .0185537 .0010926 .0165292 .020821

1 2019_11 | .019556 .0011124 .0174907 .0218597

1 2019_12 | .0191793 .0010948 .0171471 .0214471

1 2019_4 | .0186466 .0015032 .0159177 .0218329

1 2019_5 | .0186438 .001124 .0165639 .0209794

1 2019_6 | .0184949 .0011342 .0163982 .020854

1 2019_7 | .0172914 .001059 .0153337 .0194942

1 2019_8 | .0193698 .0011074 .0173144 .0216639

1 2019_9 | .018604 .0011134 .0165428 .0209166

----------------------------------------------------------------------------------

Psychosis

. proportion MEDREADS_Psychosis_Case , over(YearMonthCat)

Proportion estimation Number of obs = 128,501

--------------------------------------------------------------------------------------

| Logit

| Proportion Std. err. [95% conf. interval]

-------------------------------------+------------------------------------------------

MEDREADS_Psychosis_Case@YearMonthCat |

0 2019_10 | .9741035 .001286 .9714599 .9765081

0 2019_11 | .9736672 .0012864 .971025 .9760745

0 2019_12 | .9731107 .0012912 .9704608 .975529

0 2019_4 | .9774018 .0016515 .973928 .9804221

0 2019_5 | .9754868 .001285 .9728381 .9778831

0 2019_6 | .9762613 .0012815 .9736157 .9786475

0 2019_7 | .9770987 .0012152 .9745919 .9793634

0 2019_8 | .9766271 .001214 .9741256 .9788919

0 2019_9 | .9763036 .0012533 .9737189 .9786398

1 2019_10 | .0258965 .001286 .0234919 .0285401

1 2019_11 | .0263328 .0012864 .0239255 .028975

1 2019_12 | .0268893 .0012912 .024471 .0295392

1 2019_4 | .0225982 .0016515 .0195779 .026072

1 2019_5 | .0245132 .001285 .0221169 .0271619

1 2019_6 | .0237387 .0012815 .0213525 .0263843

1 2019_7 | .0229013 .0012152 .0206366 .0254081

1 2019_8 | .0233729 .001214 .0211081 .0258744

1 2019_9 | .0236964 .0012533 .0213602 .0262811

--------------------------------------------------------------------------------------

Substance Use

. proportion MEDREADS_Subst_Case , over(YearMonthCat)

Proportion estimation Number of obs = 128,501

----------------------------------------------------------------------------------

| Logit

| Proportion Std. err. [95% conf. interval]

---------------------------------+------------------------------------------------

MEDREADS_Subst_Case@YearMonthCat |

0 2019_10 | .8812037 .0026198 .8759722 .8862431

0 2019_11 | .8505228 .0028645 .8448211 .8560507

0 2019_12 | .8712247 .0026737 .8658928 .8763749

0 2019_4 | .883428 .0035661 .8762546 .8902377

0 2019_5 | .8880679 .0026199 .8828291 .8931009

0 2019_6 | .8894558 .0026396 .8841752 .8945243

0 2019_7 | .8835137 .0026062 .8783076 .8885254

0 2019_8 | .8813275 .0025986 .8761389 .8863269

0 2019_9 | .8794813 .0026827 .8741235 .8846411

1 2019_10 | .1187963 .0026198 .1137569 .1240278

1 2019_11 | .1494772 .0028645 .1439493 .1551789

1 2019_12 | .1287753 .0026737 .1236251 .1341072

1 2019_4 | .116572 .0035661 .1097623 .1237454

1 2019_5 | .1119321 .0026199 .1068991 .1171709

1 2019_6 | .1105442 .0026396 .1054757 .1158248

1 2019_7 | .1164863 .0026062 .1114746 .1216924

1 2019_8 | .1186725 .0025986 .1136731 .1238611

1 2019_9 | .1205187 .0026827 .1153589 .1258765

----------------------------------------------------------------------------------

. clear

(145 vars, 233,134 obs)

Suicide

. encode Year_Month, gen(YearMonthCat)

. proportion MEDREADS_Suicidal_Case, over(YearMonthCat)

Proportion estimation Number of obs = 233,134

-------------------------------------------------------------------------------------

| Logit

| Proportion Std. err. [95% conf. interval]

------------------------------------+------------------------------------------------

MEDREADS_Suicidal_Case@YearMonthCat |

0 2020_1 | .912163 .0022413 .9076698 .9164577

0 2020_10 | .9294606 .0018104 .9258286 .9329276

0 2020_11 | .9327128 .0017866 .9291255 .9361311

0 2020_12 | .9439332 .0015016 .9409164 .9468046

0 2020_2 | .9159954 .0022823 .9114127 .9203617

0 2020_3 | .9356493 .0018624 .9319011 .9392046

0 2020_4 | .9336353 .0021107 .9293767 .9376544

0 2020_5 | .9387719 .0018467 .9350509 .9422929

0 2020_6 | .9429834 .0016717 .9396171 .9461727

0 2020_7 | .9451582 .001505 .9421326 .9480344

0 2020_8 | .9546749 .0012491 .9521628 .9570611

0 2020_9 | .9405522 .0016204 .9372958 .9436497

1 2020_1 | .087837 .0022413 .0835423 .0923302

1 2020_10 | .0705394 .0018104 .0670724 .0741714

1 2020_11 | .0672872 .0017866 .0638689 .0708745

1 2020_12 | .0560668 .0015016 .0531954 .0590836

1 2020_2 | .0840046 .0022823 .0796383 .0885873

1 2020_3 | .0643507 .0018624 .0607954 .0680989

1 2020_4 | .0663647 .0021107 .0623456 .0706233

1 2020_5 | .0612281 .0018467 .0577071 .0649491

1 2020_6 | .0570166 .0016717 .0538273 .0603829

1 2020_7 | .0548418 .001505 .0519656 .0578674

1 2020_8 | .0453251 .0012491 .0429389 .0478372

1 2020_9 | .0594478 .0016204 .0563503 .0627042

-------------------------------------------------------------------------------------

EDO

. proportion MEDREADS_EDO_Case , over(YearMonthCat)

Proportion estimation Number of obs = 233,134

--------------------------------------------------------------------------------

| Logit

| Proportion Std. err. [95% conf. interval]

-------------------------------+------------------------------------------------

MEDREADS_EDO_Case@YearMonthCat |

0 2020_1 | .9971787 .00042 .9962234 .9978929

0 2020_10 | .9964505 .0004205 .9955233 .9971863

0 2020_11 | .9952701 .0004893 .9942075 .9961385

0 2020_12 | .9968473 .0003659 .9960423 .997489

0 2020_2 | .9964801 .0004873 .9953836 .9973168

0 2020_3 | .9971771 .0004027 .9962669 .9978659

0 2020_4 | .9967645 .0004815 .9956692 .9975834

0 2020_5 | .9964996 .0004549 .9954846 .997287

0 2020_6 | .9976611 .0003483 .9968689 .9982533

0 2020_7 | .9975529 .0003266 .9968215 .9981163

0 2020_8 | .9976923 .0002881 .9970527 .9981933

0 2020_9 | .9963843 .0004113 .9954817 .9971071

1 2020_1 | .0028213 .00042 .0021071 .0037766

1 2020_10 | .0035495 .0004205 .0028137 .0044767

1 2020_11 | .0047299 .0004893 .0038615 .0057925

1 2020_12 | .0031527 .0003659 .002511 .0039577

1 2020_2 | .0035199 .0004873 .0026832 .0046164

1 2020_3 | .0028229 .0004027 .0021341 .0037331

1 2020_4 | .0032355 .0004815 .0024166 .0043308

1 2020_5 | .0035004 .0004549 .002713 .0045154

1 2020_6 | .0023389 .0003483 .0017467 .0031311

1 2020_7 | .0024471 .0003266 .0018837 .0031785

1 2020_8 | .0023077 .0002881 .0018067 .0029473

1 2020_9 | .0036157 .0004113 .0028929 .0045183

--------------------------------------------------------------------------------

Mania

. proportion MEDREADS_Mania_Case , over(YearMonthCat)

Proportion estimation Number of obs = 233,134

----------------------------------------------------------------------------------

| Logit

| Proportion Std. err. [95% conf. interval]

---------------------------------+------------------------------------------------

MEDREADS_Mania_Case@YearMonthCat |

0 2020_1 | .9789342 .0011371 .9765863 .9810512

0 2020_10 | .9840024 .0008871 .9821675 .9856513

0 2020_11 | .9849964 .000867 .983199 .9866042

0 2020_12 | .9873892 .0007283 .9858789 .9887398

0 2020_2 | .9794896 .0011661 .9770749 .9816548

0 2020_3 | .9833506 .0009712 .9813362 .9851509

0 2020_4 | .9806586 .0011678 .9782319 .9828195

0 2020_5 | .983981 .000967 .9819711 .9857701

0 2020_6 | .9844075 .0008932 .9825566 .9860648

0 2020_7 | .9868904 .0007519 .9853319 .9882853

0 2020_8 | .9893989 .000615 .9881232 .9905389

0 2020_9 | .9852085 .0008272 .9834965 .9867453

1 2020_1 | .0210658 .0011371 .0189488 .0234137

1 2020_10 | .0159976 .0008871 .0143487 .0178325

1 2020_11 | .0150036 .000867 .0133958 .016801

1 2020_12 | .0126108 .0007283 .0112602 .0141211

1 2020_2 | .0205104 .0011661 .0183452 .0229251

1 2020_3 | .0166494 .0009712 .0148491 .0186638

1 2020_4 | .0193414 .0011678 .0171805 .0217681

1 2020_5 | .016019 .000967 .0142299 .0180289

1 2020_6 | .0155925 .0008932 .0139352 .0174434

1 2020_7 | .0131096 .0007519 .0117147 .0146681

1 2020_8 | .0106011 .000615 .0094611 .0118768

1 2020_9 | .0147915 .0008272 .0132547 .0165035

----------------------------------------------------------------------------------

Psychosis

. proportion MEDREADS_Psychosis_Case , over(YearMonthCat)

Proportion estimation Number of obs = 233,134

--------------------------------------------------------------------------------------

| Logit

| Proportion Std. err. [95% conf. interval]

-------------------------------------+------------------------------------------------

MEDREADS_Psychosis_Case@YearMonthCat |

0 2020_1 | .9729154 .0012853 .9702789 .9753239

0 2020_10 | .9771034 .0010576 .9749364 .9790871

0 2020_11 | .9791476 .001019 .9770538 .981054

0 2020_12 | .9832566 .0008375 .9815336 .9848214

0 2020_2 | .9733974 .001324 .9706757 .9758728

0 2020_3 | .9777624 .0011192 .9754598 .9798535

0 2020_4 | .9754817 .0013114 .9727758 .9779248

0 2020_5 | .9774548 .0011434 .9751014 .9795903

0 2020_6 | .9798857 .0010121 .9778031 .9817765

0 2020_7 | .9811659 .0008986 .9793214 .9828487

0 2020_8 | .9828363 .0007799 .9812389 .9842999

0 2020_9 | .9778832 .0010078 .9758194 .9797745

1 2020_1 | .0270846 .0012853 .0246761 .0297211

1 2020_10 | .0228966 .0010576 .0209129 .0250636

1 2020_11 | .0208524 .001019 .018946 .0229462

1 2020_12 | .0167434 .0008375 .0151786 .0184664

1 2020_2 | .0266026 .001324 .0241272 .0293243

1 2020_3 | .0222376 .0011192 .0201465 .0245402

1 2020_4 | .0245183 .0013114 .0220752 .0272242

1 2020_5 | .0225452 .0011434 .0204097 .0248986

1 2020_6 | .0201143 .0010121 .0182235 .0221969

1 2020_7 | .0188341 .0008986 .0171513 .0206786

1 2020_8 | .0171637 .0007799 .0157001 .0187611

1 2020_9 | .0221168 .0010078 .0202255 .0241806

--------------------------------------------------------------------------------------

Substance Use

. proportion MEDREADS_Subst_Case , over(YearMonthCat)

Proportion estimation Number of obs = 233,134

----------------------------------------------------------------------------------

| Logit

| Proportion Std. err. [95% conf. interval]

---------------------------------+------------------------------------------------

MEDREADS_Subst_Case@YearMonthCat |

0 2020_1 | .8714734 .00265 .8661894 .8765784

0 2020_10 | .8951157 .0021664 .8907931 .8992867

0 2020_11 | .8936019 .002199 .8892144 .8978356

0 2020_12 | .9112134 .0018566 .9075066 .9147855

0 2020_2 | .8701009 .002766 .8645827 .8754266

0 2020_3 | .9018896 .0022578 .8973746 .9062268

0 2020_4 | .9015674 .002526 .8965044 .9064087

0 2020_5 | .9008603 .0023019 .8962563 .9052815

0 2020_6 | .9115385 .0020472 .907443 .9154696

0 2020_7 | .9165793 .0018279 .912926 .9200927

0 2020_8 | .9225471 .0016051 .919342 .9256352

0 2020_9 | .900263 .0020534 .8961656 .9042159

1 2020_1 | .1285266 .00265 .1234216 .1338106

1 2020_10 | .1048843 .0021664 .1007133 .1092069

1 2020_11 | .1063981 .002199 .1021644 .1107856

1 2020_12 | .0887866 .0018566 .0852145 .0924934

1 2020_2 | .1298991 .002766 .1245734 .1354173

1 2020_3 | .0981104 .0022578 .0937732 .1026254

1 2020_4 | .0984326 .002526 .0935913 .1034956

1 2020_5 | .0991397 .0023019 .0947185 .1037437

1 2020_6 | .0884615 .0020472 .0845304 .092557

1 2020_7 | .0834207 .0018279 .0799073 .087074

1 2020_8 | .0774529 .0016051 .0743648 .080658

1 2020_9 | .099737 .0020534 .0957841 .1038344

----------------------------------------------------------------------------------

. clear

(145 vars, 328,964 obs)

. encode Year_Month, gen(YearMonthCat)

Suicide

. proportion MEDREADS_Suicidal_Case, over(YearMonthCat)

Proportion estimation Number of obs = 328,964

-------------------------------------------------------------------------------------

| Logit

| Proportion Std. err. [95% conf. interval]

------------------------------------+------------------------------------------------

MEDREADS_Suicidal_Case@YearMonthCat |

0 2021_1 | .9448927 .0013915 .9421011 .9475573

0 2021_10 | .9421142 .0016014 .9388945 .9451741

0 2021_11 | .9399292 .001644 .936625 .9430715

0 2021_12 | .9641131 .0010159 .9620678 .966052

0 2021_2 | .9378039 .0016757 .9344375 .9410083

0 2021_3 | .9381831 .001602 .9349678 .9412494

0 2021_4 | .9357965 .0017414 .9322977 .9391263

0 2021_5 | .9348074 .0017023 .9313904 .9380655

0 2021_6 | .9518876 .001427 .9490122 .9546086

0 2021_7 | .9512343 .0013679 .9484823 .9538464

0 2021_8 | .9479794 .0014782 .9450047 .9508016

0 2021_9 | .9455712 .0015526 .9424467 .9485354

0 2022_1 | .9705769 .0009189 .9687214 .9723256

0 2022_2 | .9352057 .001879 .9314239 .9387926

1 2021_1 | .0551073 .0013915 .0524427 .0578989

1 2021_10 | .0578858 .0016014 .0548259 .0611055

1 2021_11 | .0600708 .001644 .0569285 .063375

1 2021_12 | .0358869 .0010159 .033948 .0379322

1 2021_2 | .0621961 .0016757 .0589917 .0655625

1 2021_3 | .0618169 .001602 .0587506 .0650322

1 2021_4 | .0642035 .0017414 .0608737 .0677023

1 2021_5 | .0651926 .0017023 .0619345 .0686096

1 2021_6 | .0481124 .001427 .0453914 .0509878

1 2021_7 | .0487657 .0013679 .0461536 .0515177

1 2021_8 | .0520206 .0014782 .0491984 .0549953

1 2021_9 | .0544288 .0015526 .0514646 .0575533

1 2022_1 | .0294231 .0009189 .0276744 .0312786

1 2022_2 | .0647943 .001879 .0612074 .0685761

-------------------------------------------------------------------------------------

EDO

. proportion MEDREADS_EDO_Case , over(YearMonthCat)

Proportion estimation Number of obs = 328,964

--------------------------------------------------------------------------------

| Logit

| Proportion Std. err. [95% conf. interval]

-------------------------------+------------------------------------------------

MEDREADS_EDO_Case@YearMonthCat |

0 2021_1 | .9970624 .00033 .9963392 .9976432

0 2021_10 | .9967084 .0003928 .9958415 .997395

0 2021_11 | .99574 .0004506 .9947591 .9965379

0 2021_12 | .997882 .0002511 .9973281 .9983212

0 2021_2 | .9960044 .0004377 .995048 .9967767

0 2021_3 | .9969025 .0003696 .9960866 .9975487

0 2021_4 | .9964668 .0004216 .9955364 .9972038

0 2021_5 | .9967665 .0003915 .9959009 .9974498

0 2021_6 | .9972876 .0003468 .9965154 .997889

0 2021_7 | .9981042 .0002763 .9974777 .9985753

0 2021_8 | .996854 .0003728 .9960319 .9975061

0 2021_9 | .9961122 .0004259 .9951815 .9968637

0 2022_1 | .9976935 .0002609 .9971213 .9981521

0 2022_2 | .9962708 .0004653 .9952383 .9970801

1 2021_1 | .0029376 .00033 .0023568 .0036608

1 2021_10 | .0032916 .0003928 .002605 .0041585

1 2021_11 | .00426 .0004506 .0034621 .0052409

1 2021_12 | .002118 .0002511 .0016788 .0026719

1 2021_2 | .0039956 .0004377 .0032233 .004952

1 2021_3 | .0030975 .0003696 .0024513 .0039134

1 2021_4 | .0035332 .0004216 .0027962 .0044636

1 2021_5 | .0032335 .0003915 .0025502 .0040991

1 2021_6 | .0027124 .0003468 .002111 .0034846

1 2021_7 | .0018958 .0002763 .0014247 .0025223

1 2021_8 | .003146 .0003728 .0024939 .0039681

1 2021_9 | .0038878 .0004259 .0031363 .0048185

1 2022_1 | .0023065 .0002609 .0018479 .0028787

1 2022_2 | .0037292 .0004653 .0029199 .0047617

--------------------------------------------------------------------------------

Mania

. proportion MEDREADS_Mania_Case , over(YearMonthCat)

Proportion estimation Number of obs = 328,964

----------------------------------------------------------------------------------

| Logit

| Proportion Std. err. [95% conf. interval]

---------------------------------+------------------------------------------------

MEDREADS_Mania_Case@YearMonthCat |

0 2021_1 | .9885844 .0006478 .9872424 .9897867

0 2021_10 | .9873037 .0007678 .9857074 .9887237

0 2021_11 | .9869807 .0007843 .9853505 .9884315

0 2021_12 | .9924527 .0004727 .9914675 .993325

0 2021_2 | .9862321 .0008085 .9845542 .98773

0 2021_3 | .9858401 .0007859 .9842141 .9873007

0 2021_4 | .9860186 .0008342 .9842858 .9875627

0 2021_5 | .9866381 .0007918 .984994 .9881043

0 2021_6 | .9871048 .0007523 .9855439 .9884991

0 2021_7 | .9867296 .0007268 .9852271 .9880811

0 2021_8 | .9886565 .0007049 .9871883 .9899582

0 2021_9 | .9870252 .0007745 .9854161 .9884587

0 2022_1 | .9931395 .0004489 .9922013 .9939656

0 2022_2 | .985666 .0009073 .9837745 .9873398

1 2021_1 | .0114156 .0006478 .0102133 .0127576

1 2021_10 | .0126963 .0007678 .0112763 .0142926

1 2021_11 | .0130193 .0007843 .0115685 .0146495

1 2021_12 | .0075473 .0004727 .006675 .0085325

1 2021_2 | .0137679 .0008085 .01227 .0154458

1 2021_3 | .0141599 .0007859 .0126993 .0157859

1 2021_4 | .0139814 .0008342 .0124373 .0157142

1 2021_5 | .0133619 .0007918 .0118957 .015006

1 2021_6 | .0128952 .0007523 .0115009 .0144561

1 2021_7 | .0132704 .0007268 .0119189 .0147729

1 2021_8 | .0113435 .0007049 .0100418 .0128117

1 2021_9 | .0129748 .0007745 .0115413 .0145839

1 2022_1 | .0068605 .0004489 .0060344 .0077987

1 2022_2 | .014334 .0009073 .0126602 .0162255

----------------------------------------------------------------------------------

Psychosis

. proportion MEDREADS_Psychosis_Case , over(YearMonthCat)

Proportion estimation Number of obs = 328,964

--------------------------------------------------------------------------------------

| Logit

| Proportion Std. err. [95% conf. interval]

-------------------------------------+------------------------------------------------

MEDREADS_Psychosis_Case@YearMonthCat |

0 2021_1 | .9831183 .0007856 .9815075 .9845909

0 2021_10 | .9792627 .0009772 .9772583 .9810938

0 2021_11 | .9791786 .0009879 .9771517 .9810292

0 2021_12 | .986934 .0006202 .9856608 .9880954

0 2021_2 | .9814663 .0009358 .9795404 .9832141

0 2021_3 | .9794681 .0009433 .9775354 .9812378

0 2021_4 | .9783969 .0010329 .976277 .9803312

0 2021_5 | .9767 .0010403 .9745716 .9786541

0 2021_6 | .9808351 .0009143 .9789586 .9825472

0 2021_7 | .9819297 .000846 .9801947 .9835152

0 2021_8 | .9816554 .0008933 .9798201 .9833267

0 2021_9 | .9797649 .0009637 .9777872 .9815698

0 2022_1 | .990005 .0005409 .9888872 .9910114

0 2022_2 | .9801888 .0010637 .9779928 .9821696

1 2021_1 | .0168817 .0007856 .0154091 .0184925

1 2021_10 | .0207373 .0009772 .0189062 .0227417

1 2021_11 | .0208214 .0009879 .0189708 .0228483

1 2021_12 | .013066 .0006202 .0119046 .0143392

1 2021_2 | .0185337 .0009358 .0167859 .0204596

1 2021_3 | .0205319 .0009433 .0187622 .0224646

1 2021_4 | .0216031 .0010329 .0196688 .023723

1 2021_5 | .0233 .0010403 .0213459 .0254284

1 2021_6 | .0191649 .0009143 .0174528 .0210414

1 2021_7 | .0180703 .000846 .0164848 .0198053

1 2021_8 | .0183446 .0008933 .0166733 .0201799

1 2021_9 | .0202351 .0009637 .0184302 .0222128

1 2022_1 | .009995 .0005409 .0089886 .0111128

1 2022_2 | .0198112 .0010637 .0178304 .0220072

--------------------------------------------------------------------------------------

Substance Use

. proportion MEDREADS_Subst_Case , over(YearMonthCat)

Proportion estimation Number of obs = 328,964

----------------------------------------------------------------------------------

| Logit

| Proportion Std. err. [95% conf. interval]

---------------------------------+------------------------------------------------

MEDREADS_Subst_Case@YearMonthCat |

0 2021_1 | .9172275 .0016802 .9138742 .9204616

0 2021_10 | .9069407 .0019922 .9029619 .9107724

0 2021_11 | .8997702 .0020777 .895624 .9037695

0 2021_12 | .9412923 .0012839 .9387246 .9437588

0 2021_2 | .9046358 .0020379 .9005661 .9085559

0 2021_3 | .9039781 .0019598 .9000676 .9077512

0 2021_4 | .9022814 .0021096 .898068 .9063389

0 2021_5 | .9002378 .0020665 .8961137 .9042156

0 2021_6 | .9125795 .0018835 .9088168 .9162012

0 2021_7 | .9171507 .0017507 .9136541 .920518

0 2021_8 | .9143034 .0018633 .9105802 .9178856

0 2021_9 | .9106281 .0019525 .9067268 .9143817

0 2022_1 | .9526569 .0011549 .9503413 .9548697

0 2022_2 | .9010022 .0022798 .8964433 .9053816

1 2021_1 | .0827725 .0016802 .0795384 .0861258

1 2021_10 | .0930593 .0019922 .0892276 .0970381

1 2021_11 | .1002298 .0020777 .0962305 .104376

1 2021_12 | .0587077 .0012839 .0562412 .0612754

1 2021_2 | .0953642 .0020379 .0914441 .0994339

1 2021_3 | .0960219 .0019598 .0922488 .0999324

1 2021_4 | .0977186 .0021096 .0936611 .101932

1 2021_5 | .0997622 .0020665 .0957844 .1038863

1 2021_6 | .0874205 .0018835 .0837988 .0911832

1 2021_7 | .0828493 .0017507 .079482 .0863459

1 2021_8 | .0856966 .0018633 .0821144 .0894198

1 2021_9 | .0893719 .0019525 .0856183 .0932732

1 2022_1 | .0473431 .0011549 .0451303 .0496587

1 2022_2 | .0989978 .0022798 .0946184 .1035567

----------------------------------------------------------------------------------

Calculating alpha and beta parameters for Beta distribution for prevalence prior. (https://epitools.ausvet.com.au/betaparamsone)

Suicide

. proportion RaterRatedSuicidalPresentati

Proportion estimation Number of obs = 39,883

------------------------------------------------------------------------------

| Logit

| Proportion Std. err. [95% conf. interval]

-----------------------------+------------------------------------------------

RaterRatedSuicidalPresentati |

0 | .9707645 .0008436 .9690649 .9723733

1 | .0292355 .0008436 .0276267 .0309351

------------------------------------------------------------------------------

.

Estimate parameters for Beta distributions from mode & 5/95th percentile

Analysed: Thu Apr 28, 2022 @ 06:42 UTC

Results

Distribution summary

| \|  \| **Mode** \| **5/95 percentile** \| **Alpha value** \| **Beta value** \| **2.5% percentile** \| **5% percentile** \| **25% percentile** \| **50% percentile** \| **75% percentile** \| **95% percentile** \| **97.5% percentile** \| **Mean** \| **Mode** \| **Standard deviation** \| \| --- \| --- \| --- \| --- \| --- \| --- \| --- \| --- \| --- \| --- \| --- \| --- \| --- \| --- \| --- \| \| Distribution 1 \| 0.0292355 \| 0.0309351 \| 828.7296 \| 27485.7551 \| 0.0273 \| 0.0276 \| 0.0286 \| 0.0293 \| 0.0299 \| 0.0309 \| 0.0313 \| 0.0293 \| 0.0292 \| 0.001 \| |
| --- | --- | --- | --- | --- | --- | --- | --- | --- | --- | --- | --- | --- | --- | --- | --- | --- | --- | --- | --- | --- | --- | --- | --- | --- | --- | --- | --- | --- | --- | --- |

. clear

EDO

. proportion EDOCase

Proportion estimation Number of obs = 39,885

--------------------------------------------------------------

| Logit

| Proportion Std. err. [95% conf. interval]

-------------+------------------------------------------------

EDOCase |

0 | .9988968 .0001662 .9985179 .999179

1 | .0011032 .0001662 .000821 .0014821

--------------------------------------------------------------

Estimate parameters for Beta distributions from mode & 5/95th percentile

Analysed: Thu Apr 28, 2022 @ 06:45 UTC

Results

Distribution summary

| \|  \| **Mode** \| **5/95 percentile** \| **Alpha value** \| **Beta value** \| **2.5% percentile** \| **5% percentile** \| **25% percentile** \| **50% percentile** \| **75% percentile** \| **95% percentile** \| **97.5% percentile** \| **Mean** \| **Mode** \| **Standard deviation** \| \| --- \| --- \| --- \| --- \| --- \| --- \| --- \| --- \| --- \| --- \| --- \| --- \| --- \| --- \| --- \| \| Distribution 1 \| 0.0011032 \| 0.0014821 \| 32.9112 \| 28895.106 \| 8e-04 \| 8e-04 \| 0.001 \| 0.0011 \| 0.0013 \| 0.0015 \| 0.0016 \| 0.0011 \| 0.0011 \| 2e-04 \| |
| --- | --- | --- | --- | --- | --- | --- | --- | --- | --- | --- | --- | --- | --- | --- | --- | --- | --- | --- | --- | --- | --- | --- | --- | --- | --- | --- | --- | --- | --- | --- |

. clear

Mania

. proportion ManiaCase

Proportion estimation Number of obs = 39,885

--------------------------------------------------------------

| Logit

| Proportion Std. err. [95% conf. interval]

-------------+------------------------------------------------

ManiaCase |

0 | .9943839 .0003742 .9936007 .9950716

1 | .0056161 .0003742 .0049284 .0063993

--------------------------------------------------------------

Estimate parameters for Beta distributions from mode & 5/95th percentile

Analysed: Thu Apr 28, 2022 @ 06:49 UTC

Results

Distribution summary

| \|  \| **Mode** \| **5/95 percentile** \| **Alpha value** \| **Beta value** \| **2.5% percentile** \| **5% percentile** \| **25% percentile** \| **50% percentile** \| **75% percentile** \| **95% percentile** \| **97.5% percentile** \| **Mean** \| **Mode** \| **Standard deviation** \| \| --- \| --- \| --- \| --- \| --- \| --- \| --- \| --- \| --- \| --- \| --- \| --- \| --- \| --- \| --- \| \| Distribution 1 \| 0.0056161 \| 0.0063993 \| 161.4339 \| 28407.3433 \| 0.0048 \| 0.0049 \| 0.0053 \| 0.0056 \| 0.0059 \| 0.0064 \| 0.0066 \| 0.0057 \| 0.0056 \| 4e-04 \| |
| --- | --- | --- | --- | --- | --- | --- | --- | --- | --- | --- | --- | --- | --- | --- | --- | --- | --- | --- | --- | --- | --- | --- | --- | --- | --- | --- | --- | --- | --- | --- |

. clear

Psychosis

. proportion PsychosisCase

Proportion estimation Number of obs = 39,885

---------------------------------------------------------------

| Logit

| Proportion Std. err. [95% conf. interval]

--------------+------------------------------------------------

PsychosisCase |

0 | .9854582 .0005994 .9842354 .9865875

1 | .0145418 .0005994 .0134125 .0157646

---------------------------------------------------------------

Estimate parameters for Beta distributions from mode & 5/95th percentile

Analysed: Thu Apr 28, 2022 @ 06:51 UTC

Results

Distribution summary

| \|  \| **Mode** \| **5/95 percentile** \| **Alpha value** \| **Beta value** \| **2.5% percentile** \| **5% percentile** \| **25% percentile** \| **50% percentile** \| **75% percentile** \| **95% percentile** \| **97.5% percentile** \| **Mean** \| **Mode** \| **Standard deviation** \| \| --- \| --- \| --- \| --- \| --- \| --- \| --- \| --- \| --- \| --- \| --- \| --- \| --- \| --- \| --- \| \| Distribution 1 \| 0.0145418 \| 0.0157646 \| 414.1162 \| 27996.7619 \| 0.0132 \| 0.0134 \| 0.0141 \| 0.0146 \| 0.015 \| 0.0158 \| 0.016 \| 0.0146 \| 0.0145 \| 7e-04 \| |
| --- | --- | --- | --- | --- | --- | --- | --- | --- | --- | --- | --- | --- | --- | --- | --- | --- | --- | --- | --- | --- | --- | --- | --- | --- | --- | --- | --- | --- | --- | --- |

. clear

Substance Use

. proportion Subtanceuse

Proportion estimation Number of obs = 39,885

--------------------------------------------------------------

| Logit

| Proportion Std. err. [95% conf. interval]

-------------+------------------------------------------------

Subtanceuse |

0 | .9712423 .0008368 .9695559 .9728379

1 | .0287577 .0008368 .0271621 .0304441

--------------------------------------------------------------

.

Estimate parameters for Beta distributions from mode & 5/95th percentile

Analysed: Thu Apr 28, 2022 @ 06:53 UTC

Results

Distribution summary

| \|  \| **Mode** \| **5/95 percentile** \| **Alpha value** \| **Beta value** \| **2.5% percentile** \| **5% percentile** \| **25% percentile** \| **50% percentile** \| **75% percentile** \| **95% percentile** \| **97.5% percentile** \| **Mean** \| **Mode** \| **Standard deviation** \| \| --- \| --- \| --- \| --- \| --- \| --- \| --- \| --- \| --- \| --- \| --- \| --- \| --- \| --- \| --- \| \| Distribution 1 \| 0.0287577 \| 0.0304441 \| 815.3421 \| 27504.0156 \| 0.0269 \| 0.0272 \| 0.0281 \| 0.0288 \| 0.0295 \| 0.0304 \| 0.0308 \| 0.0288 \| 0.0288 \| 0.001 \| |
| --- | --- | --- | --- | --- | --- | --- | --- | --- | --- | --- | --- | --- | --- | --- | --- | --- | --- | --- | --- | --- | --- | --- | --- | --- | --- | --- | --- | --- | --- | --- |

Calculating alpha and beta parameters for Beta distribution for Sensitivity and Specificity priors. (<https://epitools.ausvet.com.au/betaparamsone>)

EDO

Sensitivity

. cii proportions 44 44

Binomial exact

Variable | Obs Proportion Std. err. [95% conf. interval]

-------------+---------------------------------------------------------------

| 44 1 0 .9195801 1*

(*) one-sided, 97.5% confidence interval

### Distribution summary

| \|  \| **Mode** \| **5/95 percentile** \| **Alpha value** \| **Beta value** \| **2.5% percentile** \| **5% percentile** \| **25% percentile** \| **50% percentile** \| **75% percentile** \| **95% percentile** \| **97.5% percentile** \| **Mean** \| **Mode** \| **Standard deviation** \| \| --- \| --- \| --- \| --- \| --- \| --- \| --- \| --- \| --- \| --- \| --- \| --- \| --- \| --- \| --- \| \| Distribution 1 \| 0.999999999999 \| 0.9195801 \| 35.7324 \| 1 \| 0.9019 \| 0.9196 \| 0.9619 \| 0.9808 \| 0.992 \| 0.9986 \| 0.9993 \| 0.9728 \| 1 \| 0.0265 \| |
| --- | --- | --- | --- | --- | --- | --- | --- | --- | --- | --- | --- | --- | --- | --- | --- | --- | --- | --- | --- | --- | --- | --- | --- | --- | --- | --- | --- | --- | --- | --- |

Specificity

. cii proportions 40008 39885

Binomial exact

Variable | Obs Proportion Std. err. [95% conf. interval]

-------------+---------------------------------------------------------------

| 40,008 .9969256 .0002768 .9963329 .9974443

### Distribution summary

| \|  \| **Mode** \| **5/95 percentile** \| **Alpha value** \| **Beta value** \| **2.5% percentile** \| **5% percentile** \| **25% percentile** \| **50% percentile** \| **75% percentile** \| **95% percentile** \| **97.5% percentile** \| **Mean** \| **Mode** \| **Standard deviation** \| \| --- \| --- \| --- \| --- \| --- \| --- \| --- \| --- \| --- \| --- \| --- \| --- \| --- \| --- \| --- \| \| Distribution 1 \| 0.9969256 \| 0.9963329 \| 28735.0476 \| 89.6124 \| 0.9962 \| 0.9963 \| 0.9967 \| 0.9969 \| 0.9971 \| 0.9974 \| 0.9975 \| 0.9969 \| 0.9969 \| 3e-04 \| |
| --- | --- | --- | --- | --- | --- | --- | --- | --- | --- | --- | --- | --- | --- | --- | --- | --- | --- | --- | --- | --- | --- | --- | --- | --- | --- | --- | --- | --- | --- | --- |

Mania

. *Sensitivity

. cii proportions 224 185

Binomial exact

Variable | Obs Proportion Std. err. [95% conf. interval]

-------------+---------------------------------------------------------------

| 224 .8258929 .0253365 .7697673 .8731666

### Distribution summary

| \|  \| **Mode** \| **5/95 percentile** \| **Alpha value** \| **Beta value** \| **2.5% percentile** \| **5% percentile** \| **25% percentile** \| **50% percentile** \| **75% percentile** \| **95% percentile** \| **97.5% percentile** \| **Mean** \| **Mode** \| **Standard deviation** \| \| --- \| --- \| --- \| --- \| --- \| --- \| --- \| --- \| --- \| --- \| --- \| --- \| --- \| --- \| --- \| \| Distribution 1 \| 0.8258929 \| 0.7697673 \| 130.5007 \| 28.3001 \| 0.7588 \| 0.7698 \| 0.8021 \| 0.8231 \| 0.843 \| 0.8692 \| 0.8771 \| 0.8218 \| 0.8259 \| 0.0303 \| |
| --- | --- | --- | --- | --- | --- | --- | --- | --- | --- | --- | --- | --- | --- | --- | --- | --- | --- | --- | --- | --- | --- | --- | --- | --- | --- | --- | --- | --- | --- | --- |

. *Specificity

. cii proportions 39661 37566

Binomial exact

Variable | Obs Proportion Std. err. [95% conf. interval]

-------------+---------------------------------------------------------------

| 39,661 .9471773 .0011232 .9449304 .9493585

### Distribution summary

| \|  \| **Mode** \| **5/95 percentile** \| **Alpha value** \| **Beta value** \| **2.5% percentile** \| **5% percentile** \| **25% percentile** \| **50% percentile** \| **75% percentile** \| **95% percentile** \| **97.5% percentile** \| **Mean** \| **Mode** \| **Standard deviation** \| \| --- \| --- \| --- \| --- \| --- \| --- \| --- \| --- \| --- \| --- \| --- \| --- \| --- \| --- \| --- \| \| Distribution 1 \| 0.9471773 \| 0.9449304 \| 26575.1465 \| 1483.0015 \| 0.9445 \| 0.9449 \| 0.9463 \| 0.9472 \| 0.9481 \| 0.9493 \| 0.9497 \| 0.9471 \| 0.9472 \| 0.0013 \| |
| --- | --- | --- | --- | --- | --- | --- | --- | --- | --- | --- | --- | --- | --- | --- | --- | --- | --- | --- | --- | --- | --- | --- | --- | --- | --- | --- | --- | --- | --- | --- |

Psychosis

. *Sensitivity

. cii proportions 580 580

Binomial exact

Variable | Obs Proportion Std. err. [95% conf. interval]

-------------+---------------------------------------------------------------

| 580 1 0 .99366 1*

(*) one-sided, 97.5% confidence interval

### Distribution summary

| \|  \| **Mode** \| **5/95 percentile** \| **Alpha value** \| **Beta value** \| **2.5% percentile** \| **5% percentile** \| **25% percentile** \| **50% percentile** \| **75% percentile** \| **95% percentile** \| **97.5% percentile** \| **Mean** \| **Mode** \| **Standard deviation** \| \| --- \| --- \| --- \| --- \| --- \| --- \| --- \| --- \| --- \| --- \| --- \| --- \| --- \| --- \| --- \| \| Distribution 1 \| 0.99999999999 \| 0.99366 \| 471.0135 \| 1 \| 0.9922 \| 0.9937 \| 0.9971 \| 0.9985 \| 0.9994 \| 0.9999 \| 0.9999 \| 0.9979 \| 1 \| 0.0021 \| |
| --- | --- | --- | --- | --- | --- | --- | --- | --- | --- | --- | --- | --- | --- | --- | --- | --- | --- | --- | --- | --- | --- | --- | --- | --- | --- | --- | --- | --- | --- | --- |

. *Specificity

. cii proportions 39305 38830

Binomial exact

Variable | Obs Proportion Std. err. [95% conf. interval]

-------------+---------------------------------------------------------------

| 39,305 .987915 .0005511 .9867853 .9889718

### Distribution summary

| \|  \| **Mode** \| **5/95 percentile** \| **Alpha value** \| **Beta value** \| **2.5% percentile** \| **5% percentile** \| **25% percentile** \| **50% percentile** \| **75% percentile** \| **95% percentile** \| **97.5% percentile** \| **Mean** \| **Mode** \| **Standard deviation** \| \| --- \| --- \| --- \| --- \| --- \| --- \| --- \| --- \| --- \| --- \| --- \| --- \| --- \| --- \| --- \| \| Distribution 1 \| 0.987915 \| 0.9867853 \| 27668.8136 \| 339.4558 \| 0.9866 \| 0.9868 \| 0.9874 \| 0.9879 \| 0.9883 \| 0.9889 \| 0.9891 \| 0.9879 \| 0.9879 \| 7e-04 \| |
| --- | --- | --- | --- | --- | --- | --- | --- | --- | --- | --- | --- | --- | --- | --- | --- | --- | --- | --- | --- | --- | --- | --- | --- | --- | --- | --- | --- | --- | --- | --- |

Substance Use

Sensitivity

. cii prop 1147 1015

Binomial exact

Variable | Obs Proportion Std. err. [95% conf. interval]

-------------+---------------------------------------------------------------

| 1,147 .8849172 .0094227 .8650185 .9028158

### Distribution summary

| \|  \| **Mode** \| **5/95 percentile** \| **Alpha value** \| **Beta value** \| **2.5% percentile** \| **5% percentile** \| **25% percentile** \| **50% percentile** \| **75% percentile** \| **95% percentile** \| **97.5% percentile** \| **Mean** \| **Mode** \| **Standard deviation** \| \| --- \| --- \| --- \| --- \| --- \| --- \| --- \| --- \| --- \| --- \| --- \| --- \| --- \| --- \| --- \| \| Distribution 1 \| 0.8849172 \| 0.8650185 \| 721.0263 \| 94.6389 \| 0.8611 \| 0.865 \| 0.8766 \| 0.8843 \| 0.8917 \| 0.9019 \| 0.905 \| 0.884 \| 0.8849 \| 0.0112 \| |
| --- | --- | --- | --- | --- | --- | --- | --- | --- | --- | --- | --- | --- | --- | --- | --- | --- | --- | --- | --- | --- | --- | --- | --- | --- | --- | --- | --- | --- | --- | --- |

. display 4145 + 34593

38738

. *Speificity

. cii prop 38738 34593

Binomial exact

Variable | Obs Proportion Std. err. [95% conf. interval]

-------------+---------------------------------------------------------------

| 38,738 .8929991 .0015705 .8898785 .8960607

### Distribution summary

| \|  \| **Mode** \| **5/95 percentile** \| **Alpha value** \| **Beta value** \| **2.5% percentile** \| **5% percentile** \| **25% percentile** \| **50% percentile** \| **75% percentile** \| **95% percentile** \| **97.5% percentile** \| **Mean** \| **Mode** \| **Standard deviation** \| \| --- \| --- \| --- \| --- \| --- \| --- \| --- \| --- \| --- \| --- \| --- \| --- \| --- \| --- \| --- \| \| Distribution 1 \| 0.8929991 \| 0.8898785 \| 24409.7377 \| 2925.7027 \| 0.8893 \| 0.8899 \| 0.8917 \| 0.893 \| 0.8942 \| 0.896 \| 0.8966 \| 0.893 \| 0.893 \| 0.0019 \| |
| --- | --- | --- | --- | --- | --- | --- | --- | --- | --- | --- | --- | --- | --- | --- | --- | --- | --- | --- | --- | --- | --- | --- | --- | --- | --- | --- | --- | --- | --- | --- |

Suicidal Cases

Sensitivity

. cii prop 1166 1135

Binomial exact

Variable | Obs Proportion Std. err. [95% conf. interval]

-------------+---------------------------------------------------------------

| 1,166 .9734134 .0047112 .9624734 .9818658

### Distribution summary

| \|  \| **Mode** \| **5/95 percentile** \| **Alpha value** \| **Beta value** \| **2.5% percentile** \| **5% percentile** \| **25% percentile** \| **50% percentile** \| **75% percentile** \| **95% percentile** \| **97.5% percentile** \| **Mean** \| **Mode** \| **Standard deviation** \| \| --- \| --- \| --- \| --- \| --- \| --- \| --- \| --- \| --- \| --- \| --- \| --- \| --- \| --- \| --- \| \| Distribution 1 \| 0.9734134 \| 0.9624734 \| 828.521 \| 23.6019 \| 0.9603 \| 0.9625 \| 0.9687 \| 0.9727 \| 0.9763 \| 0.9809 \| 0.9822 \| 0.9723 \| 0.9734 \| 0.0056 \| |
| --- | --- | --- | --- | --- | --- | --- | --- | --- | --- | --- | --- | --- | --- | --- | --- | --- | --- | --- | --- | --- | --- | --- | --- | --- | --- | --- | --- | --- | --- | --- |

. display 1698+37019

38717

Specificity

. cii prop 38717 37019

Binomial exact

Variable | Obs Proportion Std. err. [95% conf. interval]

-------------+---------------------------------------------------------------

| 38,717 .9561433 .0010407 .9540561 .9581617

.

### Distribution summary

| \|  \| **Mode** \| **5/95 percentile** \| **Alpha value** \| **Beta value** \| **2.5% percentile** \| **5% percentile** \| **25% percentile** \| **50% percentile** \| **75% percentile** \| **95% percentile** \| **97.5% percentile** \| **Mean** \| **Mode** \| **Standard deviation** \| \| --- \| --- \| --- \| --- \| --- \| --- \| --- \| --- \| --- \| --- \| --- \| --- \| --- \| --- \| --- \| \| Distribution 1 \| 0.9561433 \| 0.9540561 \| 26207.1353 \| 1203.0318 \| 0.9537 \| 0.9541 \| 0.9553 \| 0.9561 \| 0.957 \| 0.9581 \| 0.9585 \| 0.9561 \| 0.9561 \| 0.0012 \| |
| --- | --- | --- | --- | --- | --- | --- | --- | --- | --- | --- | --- | --- | --- | --- | --- | --- | --- | --- | --- | --- | --- | --- | --- | --- | --- | --- | --- | --- | --- | --- |

Observed MEDREADS Prevalence Estimates 2019

. tabstat MEDREADS_Suicidal_Case, by(Month) stats(n sum mean)

Summary for variables: MEDREADS_Suicidal_Case

Group variable: Month (Month)

Month | N Sum Mean

---------+------------------------------

4 | 8098 610 .0753272

5 | 14482 1034 .071399

6 | 14112 1002 .0710034

7 | 15152 1024 .0675818

8 | 15488 1102 .0711519

9 | 14728 1136 .077132

10 | 15253 1234 .0809021

11 | 15494 1394 .0899703

12 | 15694 1289 .0821333

---------+------------------------------

Total | 128501 9825 .0764585

----------------------------------------

. tabstat MEDREADS_EDO_Case , by(Month) stats(n sum mean)

Summary for variables: MEDREADS_EDO_Case

Group variable: Month (Month)

Month | N Sum Mean

---------+------------------------------

4 | 8098 19 .0023463

5 | 14482 35 .0024168

6 | 14112 48 .0034014

7 | 15152 53 .0034979

8 | 15488 49 .0031637

9 | 14728 57 .0038702

10 | 15253 53 .0034747

11 | 15494 51 .0032916

12 | 15694 50 .0031859

---------+------------------------------

Total | 128501 415 .0032295

----------------------------------------

. tabstat MEDREADS_Mania_Case , by(Month) stats(n sum mean)

Summary for variables: MEDREADS_Mania_Case

Group variable: Month (Month)

Month | N Sum Mean

---------+------------------------------

4 | 8098 151 .0186466

5 | 14482 270 .0186438

6 | 14112 261 .0184949

7 | 15152 262 .0172914

8 | 15488 300 .0193698

9 | 14728 274 .018604

10 | 15253 283 .0185537

11 | 15494 303 .019556

12 | 15694 301 .0191793

---------+------------------------------

Total | 128501 2405 .0187158

----------------------------------------

. tabstat MEDREADS_Psychosis_Case , by(Month) stats(n sum mean)

Summary for variables: MEDREADS_Psychosis_Case

Group variable: Month (Month)

Month | N Sum Mean

---------+------------------------------

4 | 8098 183 .0225982

5 | 14482 355 .0245132

6 | 14112 335 .0237387

7 | 15152 347 .0229013

8 | 15488 362 .0233729

9 | 14728 349 .0236964

10 | 15253 395 .0258965

11 | 15494 408 .0263328

12 | 15694 422 .0268893

---------+------------------------------

Total | 128501 3156 .0245601

----------------------------------------

. tabstat MEDREADS_Subst_Case , by(Month) stats(n sum mean)

Summary for variables: MEDREADS_Subst_Case

Group variable: Month (Month)

Month | N Sum Mean

---------+------------------------------

4 | 8098 944 .116572

5 | 14482 1621 .1119321

6 | 14112 1560 .1105442

7 | 15152 1765 .1164863

8 | 15488 1838 .1186725

9 | 14728 1775 .1205187

10 | 15253 1812 .1187963

11 | 15494 2316 .1494772

12 | 15694 2021 .1287753

---------+------------------------------

Total | 128501 15652 .1218045

----------------------------------------

.

Observed MEDREADS Prevalence Estimates 2020

. tabstat MEDREADS_Suicidal_Case, by(Month) stats(n sum mean)

Summary for variables: MEDREADS_Suicidal_Case

Group variable: Month (Month)

Month | N Sum Mean

---------+------------------------------

1 | 15950 1401 .087837

2 | 14773 1241 .0840046

3 | 17358 1117 .0643507

4 | 13908 923 .0663647

5 | 16855 1032 .0612281

6 | 19240 1097 .0570166

7 | 22884 1255 .0548418

8 | 27733 1257 .0453251

9 | 21296 1266 .0594478

10 | 20003 1411 .0705394

11 | 19662 1323 .0672872

12 | 23472 1316 .0560668

---------+------------------------------

Total | 233134 14639 .0627922

----------------------------------------

. tabstat MEDREADS_EDO_Case , by(Month) stats(n sum mean)

Summary for variables: MEDREADS_EDO_Case

Group variable: Month (Month)

Month | N Sum Mean

---------+------------------------------

1 | 15950 45 .0028213

2 | 14773 52 .0035199

3 | 17358 49 .0028229

4 | 13908 45 .0032355

5 | 16855 59 .0035004

6 | 19240 45 .0023389

7 | 22884 56 .0024471

8 | 27733 64 .0023077

9 | 21296 77 .0036157

10 | 20003 71 .0035495

11 | 19662 93 .0047299

12 | 23472 74 .0031527

---------+------------------------------

Total | 233134 730 .0031312

----------------------------------------

. tabstat MEDREADS_Mania_Case , by(Month) stats(n sum mean)

Summary for variables: MEDREADS_Mania_Case

Group variable: Month (Month)

Month | N Sum Mean

---------+------------------------------

1 | 15950 336 .0210658

2 | 14773 303 .0205104

3 | 17358 289 .0166494

4 | 13908 269 .0193414

5 | 16855 270 .016019

6 | 19240 300 .0155925

7 | 22884 300 .0131096

8 | 27733 294 .0106011

9 | 21296 315 .0147915

10 | 20003 320 .0159976

11 | 19662 295 .0150036

12 | 23472 296 .0126108

---------+------------------------------

Total | 233134 3587 .015386

----------------------------------------

. tabstat MEDREADS_Psychosis_Case , by(Month) stats(n sum mean)

Summary for variables: MEDREADS_Psychosis_Case

Group variable: Month (Month)

Month | N Sum Mean

---------+------------------------------

1 | 15950 432 .0270846

2 | 14773 393 .0266026

3 | 17358 386 .0222376

4 | 13908 341 .0245183

5 | 16855 380 .0225452

6 | 19240 387 .0201143

7 | 22884 431 .0188341

8 | 27733 476 .0171637

9 | 21296 471 .0221168

10 | 20003 458 .0228966

11 | 19662 410 .0208524

12 | 23472 393 .0167434

---------+------------------------------

Total | 233134 4958 .0212667

----------------------------------------

. tabstat MEDREADS_Subst_Case , by(Month) stats(n sum mean)

Summary for variables: MEDREADS_Subst_Case

Group variable: Month (Month)

Month | N Sum Mean

---------+------------------------------

1 | 15950 2050 .1285266

2 | 14773 1919 .1298991

3 | 17358 1703 .0981104

4 | 13908 1369 .0984326

5 | 16855 1671 .0991397

6 | 19240 1702 .0884615

7 | 22884 1909 .0834207

8 | 27733 2148 .0774529

9 | 21296 2124 .099737

10 | 20003 2098 .1048843

11 | 19662 2092 .1063981

12 | 23472 2084 .0887866

---------+------------------------------

Total | 233134 22869 .0980938

----------------------------------------

Observed MEDREADS Prevalence Estimates 2021

. tabstat MEDREADS_Suicidal_Case, by(Month) stats(n sum mean)

Summary for variables: MEDREADS_Suicidal_Case

Group variable: Month (Month)

Month | N Sum Mean

---------+------------------------------

1 | 60710 2477 .0408005

2 | 37935 2404 .0633716

3 | 22599 1397 .0618169

4 | 19812 1272 .0642035

5 | 21030 1371 .0651926

6 | 22489 1082 .0481124

7 | 24792 1209 .0487657

8 | 22568 1174 .0520206

9 | 21349 1162 .0544288

10 | 21266 1231 .0578858

11 | 20892 1255 .0600708

12 | 33522 1203 .0358869

---------+------------------------------

Total | 328964 17237 .0523978

----------------------------------------

. tabstat MEDREADS_EDO_Case , by(Month) stats(n sum mean)

Summary for variables: MEDREADS_EDO_Case

Group variable: Month (Month)

Month | N Sum Mean

---------+------------------------------

1 | 60710 157 .0025861

2 | 37935 147 .003875

3 | 22599 70 .0030975

4 | 19812 70 .0035332

5 | 21030 68 .0032335

6 | 22489 61 .0027124

7 | 24792 47 .0018958

8 | 22568 71 .003146

9 | 21349 83 .0038878

10 | 21266 70 .0032916

11 | 20892 89 .00426

12 | 33522 71 .002118

---------+------------------------------

Total | 328964 1004 .003052

----------------------------------------

. tabstat MEDREADS_Mania_Case , by(Month) stats(n sum mean)

Summary for variables: MEDREADS_Mania_Case

Group variable: Month (Month)

Month | N Sum Mean

---------+------------------------------

1 | 60710 539 .0088783

2 | 37935 532 .014024

3 | 22599 320 .0141599

4 | 19812 277 .0139814

5 | 21030 281 .0133619

6 | 22489 290 .0128952

7 | 24792 329 .0132704

8 | 22568 256 .0113435

9 | 21349 277 .0129748

10 | 21266 270 .0126963

11 | 20892 272 .0130193

12 | 33522 253 .0075473

---------+------------------------------

Total | 328964 3896 .0118432

----------------------------------------

. tabstat MEDREADS_Psychosis_Case , by(Month) stats(n sum mean)

Summary for variables: MEDREADS_Psychosis_Case

Group variable: Month (Month)

Month | N Sum Mean

---------+------------------------------

1 | 60710 792 .0130456

2 | 37935 725 .0191116

3 | 22599 464 .0205319

4 | 19812 428 .0216031

5 | 21030 490 .0233

6 | 22489 431 .0191649

7 | 24792 448 .0180703

8 | 22568 414 .0183446

9 | 21349 432 .0202351

10 | 21266 441 .0207373

11 | 20892 435 .0208214

12 | 33522 438 .013066

---------+------------------------------

Total | 328964 5938 .0180506

----------------------------------------

. tabstat MEDREADS_Subst_Case , by(Month) stats(n sum mean)

Summary for variables: MEDREADS_Subst_Case

Group variable: Month (Month)

Month | N Sum Mean

---------+------------------------------

1 | 60710 3827 .0630374

2 | 37935 3680 .097008

3 | 22599 2170 .0960219

4 | 19812 1936 .0977186

5 | 21030 2098 .0997622

6 | 22489 1966 .0874205

7 | 24792 2054 .0828493

8 | 22568 1934 .0856966

9 | 21349 1908 .0893719

10 | 21266 1979 .0930593

11 | 20892 2094 .1002298

12 | 33522 1968 .0587077

---------+------------------------------

Total | 328964 27614 .0839423

----------------------------------------

10/05/2022

Suicidality

4 2019

model {

Tpos ~ dbin(p, n)

p <- pi * Se + (1 - pi) * (1 - Sp)

Se ~ dbeta(828.521, 23.6019) ## Mode=0.9734134, 95% sure Se > 0.96247

Sp ~ dbeta(26207.1353, 1203.0318) ## Mode=0.9561, 95% sure Sp > 0.954

pi ~ dbeta(828.7296, 27485.7551) ## Mode=0.029, 95% sure pistar < 0.031

}

list(n=8098, Tpos=610)

list(Se=0.9734134, Sp=0.9561, pi=0.029) #Suicide 4 2019

**node mean sd MC error 2.5% median 97.5% start sample**

p 0.07202 0.001363 5.647E-6 0.06937 0.07201 0.07469 501 49500

pi 0.02965 9.727E-4 4.015E-6 0.02777 0.02965 0.0316 501 49500

5 2019

list(n=14482, Tpos=1034)

list(Se=0.9734134, Sp=0.9561, pi=0.029) #Suicide 5 2019

**node mean sd MC error 2.5% median 97.5% start sample**

p 0.07117 0.001246 5.052E-6 0.06874 0.07116 0.07364 501 50500

pi 0.02931 9.403E-4 4.448E-6 0.0275 0.02931 0.03118 501 50500

6 2019

list(n=14112, Tpos=1002)

list(Se=0.9734134, Sp=0.9561, pi=0.029) #Suicide 6 2019

**node mean sd MC error 2.5% median 97.5% start sample**

pi 0.02926 9.31E-4 4.348E-6 0.02745 0.02925 0.03111 501 49500

7 2019

list(n=15152, Tpos=1024)

list(Se=0.9734134, Sp=0.9561, pi=0.029) #Suicide 7 2019

**node mean sd MC error 2.5% median 97.5% start sample**

pi 0.02879 9.146E-4 4.331E-6 0.02702 0.02878 0.03061 501 49500

8 2019

list(n=15488, Tpos=1102)

list(Se=0.9734134, Sp=0.9561, pi=0.029) #Suicide 8 2019

**node mean sd MC error 2.5% median 97.5% start sample**

pi 0.02928 9.358E-4 4.508E-6 0.02747 0.02927 0.03113 501 49500

9 2019

list(n=14728, Tpos=1136)

list(Se=0.9734134, Sp=0.9561, pi=0.029) #Suicide 9 2019

**node mean sd MC error 2.5% median 97.5% start sample**

pi 0.0301 9.571E-4 4.306E-6 0.02825 0.03009 0.032 501 49500

10 2019

list(n=15235, Tpos=1234)

list(Se=0.9734134, Sp=0.9561, pi=0.029) #Suicide 10 2019

**node mean sd MC error 2.5% median 97.5% start sample**

pi 0.03066 9.663E-4 4.374E-6 0.02879 0.03065 0.03257 501 49500

11 2019

list(n=15494, Tpos=1394)

list(Se=0.9734134, Sp=0.9561, pi=0.029) #Suicide 11 2019

**node mean sd MC error 2.5% median 97.5% start sample**

pi 0.03193 9.979E-4 4.447E-6 0.03001 0.03192 0.03391 501 49500

12 2019

list(n=15694, Tpos=1289)

list(Se=0.9734134, Sp=0.9561, pi=0.029) #Suicide 12 2019

**node mean sd MC error 2.5% median 97.5% start sample**

pi 0.03085 9.704E-4 4.314E-6 0.02897 0.03084 0.0328 501 49500

1 2020

list(n=15950, Tpos=1401)

list(Se=0.9734134, Sp=0.9561, pi=0.029) #Suicide 1 2020

**node mean sd MC error 2.5% median 97.5% start sample**

pi 0.03168 0.001 4.396E-6 0.02974 0.03167 0.03366 501 49500

2 2020

list(n=14773, Tpos=1241)

list(Se=0.9734134, Sp=0.9561, pi=0.029) #Suicide 2 2020

**node mean sd MC error 2.5% median 97.5% start sample**

pi 0.03104 9.81E-4 4.738E-6 0.02916 0.03102 0.03299 501 49500

3 2020

list(n=17358, Tpos=1117)

list(Se=0.9734134, Sp=0.9561, pi=0.029) #Suicide 3 2020

**node mean sd MC error 2.5% median 97.5% start sample**

pi 0.02825 9.052E-4 4.461E-6 0.02651 0.02824 0.03005 501 49500

4 2020

list(n=13908, Tpos=923)

list(Se=0.9734134, Sp=0.9561, pi=0.029) #Suicide 4 2020

**node mean sd MC error 2.5% median 97.5% start sample**

pi 0.02866 9.196E-4 4.355E-6 0.02688 0.02865 0.03048 501 49500

5 2020

list(n=16855, Tpos=1032)

list(Se=0.9734134, Sp=0.9561, pi=0.029) #Suicide 5 2020

**node mean sd MC error 2.5% median 97.5% start sample**

pi 0.0278 8.93E-4 4.296E-6 0.0261 0.0278 0.02957 501 49500

6 2020

list(n=19240, Tpos=1097)

list(Se=0.9734134, Sp=0.9561, pi=0.029) #Suicide 6 2020

**node mean sd MC error 2.5% median 97.5% start sample**

pi 0.027 8.59E-4 3.793E-6 0.02535 0.027 0.0287 501 49500

7 2020

list(n=22884, Tpos=1255)

list(Se=0.9734134, Sp=0.9561, pi=0.029) #Suicide 7 2020

**node mean sd MC error 2.5% median 97.5% start sample**

pi 0.02637 8.387E-4 4.068E-6 0.02476 0.02637 0.02804 501 49500

8 2020

list(n=27733, Tpos=1257)

list(Se=0.9734134, Sp=0.9561, pi=0.029) #Suicide 8 2020

**node mean sd MC error 2.5% median 97.5% start sample**

pi 0.02418 7.659E-4 3.541E-6 0.02271 0.02417 0.02571 501 49500

9 2020

list(n=21296, Tpos=1266)

list(Se=0.9734134, Sp=0.9561, pi=0.029) #Suicide 9 2020

**node mean sd MC error 2.5% median 97.5% start sample**

pi 0.02728 8.71E-4 3.924E-6 0.0256 0.02727 0.02901 501 49500

10 2020

list(n=20003, Tpos=1411)

list(Se=0.9734134, Sp=0.9561, pi=0.029) #Suicide 10 2020

**node mean sd MC error 2.5% median 97.5% start sample**

pi 0.02918 9.195E-4 4.211E-6 0.02742 0.02917 0.03102 501 49500

11 2020

list(n=19662, Tpos=1323)

list(Se=0.9734134, Sp=0.9561, pi=0.029) #Suicide 11 2020

**node mean sd MC error 2.5% median 97.5% start sample**

pi 0.02865 9.038E-4 4.051E-6 0.02691 0.02864 0.03044 501 49500

12 2020

list(n=23472, Tpos=1316)

list(Se=0.9734134, Sp=0.9561, pi=0.029) #Suicide 12 2020

**node mean sd MC error 2.5% median 97.5% start sample**

pi 0.02656 8.407E-4 3.972E-6 0.02493 0.02655 0.02821 501 49500

1 2021

list(n=60710, Tpos=2477)

list(Se=0.9734134, Sp=0.9561, pi=0.029) #Suicide 1 2021

**node mean sd MC error 2.5% median 97.5% start sample**

pi 0.02102 6.475E-4 3.029E-6 0.01977 0.02101 0.02229 501 49500

2 2021

list(n=37935, Tpos=2404)

list(Se=0.9734134, Sp=0.9561, pi=0.029) #Suicide 2 2021

**node mean sd MC error 2.5% median 97.5% start sample**

pi 0.0275 8.438E-4 4.353E-6 0.02586 0.0275 0.02918 501 49500

3 2021

list(n=22599, Tpos=1379)

list(Se=0.9734134, Sp=0.9561, pi=0.029) #Suicide 3 2021

**node mean sd MC error 2.5% median 97.5% start sample**

pi 0.02749 8.609E-4 3.84E-6 0.02583 0.02748 0.02919 501 49500

4 2021

list(n=19812, Tpos=1272)

list(Se=0.9734134, Sp=0.9561, pi=0.029) #Suicide 4 2021

**node mean sd MC error 2.5% median 97.5% start sample**

pi 0.02814 8.889E-4 4.35E-6 0.02644 0.02813 0.0299 501 49500

5 2021

list(n=21030, Tpos=1371)

list(Se=0.9734134, Sp=0.9561, pi=0.029) #Suicide 5 2021

**node mean sd MC error 2.5% median 97.5% start sample**

pi 0.02826 8.885E-4 4.346E-6 0.02653 0.02826 0.03002 501 49500

6 2021

list(n=22489, Tpos=1082)

list(Se=0.9734134, Sp=0.9561, pi=0.029) #Suicide 6 2021

**node mean sd MC error 2.5% median 97.5% start sample**

pi 0.02521 8.056E-4 3.659E-6 0.02364 0.0252 0.02682 501 49500

7 2021

list(n=24792, Tpos=1209)

list(Se=0.9734134, Sp=0.9561, pi=0.029) #Suicide 7 2021

**node mean sd MC error 2.5% median 97.5% start sample**

pi 0.02511 8.01E-4 4.026E-6 0.02356 0.0251 0.0267 501 49500

8 2021

list(n=22568, Tpos=1174)

list(Se=0.9734134, Sp=0.9561, pi=0.029) #Suicide 8 2021

**node mean sd MC error 2.5% median 97.5% start sample**

pi 0.02589 8.274E-4 3.834E-6 0.0243 0.02589 0.02754 501 49500

9 2021

list(n=21349, Tpos=1162)

list(Se=0.9734134, Sp=0.9561, pi=0.029) #Suicide 9 2021

**node mean sd MC error 2.5% median 97.5% start sample**

pi 0.02641 8.434E-4 4.021E-6 0.02477 0.0264 0.02808 501 49500

10 2021

list(n=21266, Tpos=1231)

list(Se=0.9734134, Sp=0.9561, pi=0.029) #Suicide 10 2021

**node mean sd MC error 2.5% median 97.5% start sample**

pi 0.02702 8.579E-4 4.376E-6 0.02537 0.02701 0.02873 501 49500

11 2021

list(n=20892, Tpos=1255)

list(Se=0.9734134, Sp=0.9561, pi=0.029) #Suicide 11 2021

**node mean sd MC error 2.5% median 97.5% start sample**

pi 0.0274 8.742E-4 4.098E-6 0.02573 0.02739 0.02914 501 49500

12 2021

list(n=33522, Tpos=1203)

list(Se=0.9734134, Sp=0.9561, pi=0.029) #Suicide 12 2021

**node mean sd MC error 2.5% median 97.5% start sample**

pi 0.02164 6.901E-4 3.178E-6 0.02031 0.02164 0.02302 501 49500

EDO

4 2019

model {

Tpos ~ dbin(p, n)

p <- pi * Se + (1 - pi) * (1 - Sp)

Se ~ dbeta(35.7324, 1) ## Mode=1, 95% sure Se > 0.9195801

Sp ~ dbeta(28735.0476, 89.6124) ## Mode=0.996925, 95% sure Sp > 0.9963329

pi ~ dbeta(32.9112, 28895.106) ## Mode=0.0011032, 95% sure pi > 0.0014821

}

ist(n=8098, Tpos=19)

list(Se=.99999999, Sp=0.996925, pi=0.0011032) #EDO 4 2019

**node mean sd MC error 2.5% median 97.5% start sample**

pi 0.00103 1.764E-4 7.944E-7 7.149E-4 0.001019 0.001404 501 49500

5 2019

ist(n=14482, Tpos=35)

list(Se=.99999999, Sp=0.996925, pi=0.0011032) #EDO 5 2019

**node mean sd MC error 2.5% median 97.5% start sample**

pi 9.794E-4 1.656E-4 7.718E-7 6.827E-4 9.702E-4 0.001328 501 49500

6 2019

list(n=14112, Tpos=48)

list(Se=.99999999, Sp=0.996925, pi=0.0011032) #EDO 6 2019

**node mean sd MC error 2.5% median 97.5% start sample**

pi 0.001067 1.788E-4 7.973E-7 7.437E-4 0.001058 0.001443 501 49500

7 2019

list(n=15152, Tpos=53)

list(Se=.99999999, Sp=0.996925, pi=0.0011032) #EDO 7 2019

**node mean sd MC error 2.5% median 97.5% start sample**

pi 0.001072 1.789E-4 9.085E-7 7.495E-4 0.001061 0.00145 501 49500

8 2019

list(n=15488, Tpos=49)

list(Se=.99999999, Sp=0.996925, pi=0.0011032) #EDO 8 2019

**node mean sd MC error 2.5% median 97.5% start sample**

pi 0.001041 1.743E-4 8.429E-7 7.247E-4 0.001031 0.001407 501 49500

9 2019

list(n=14728, Tpos=57)

list(Se=.99999999, Sp=0.996925, pi=0.0011032) #EDO 9 2019

**node mean sd MC error 2.5% median 97.5% start sample**

pi 0.001106 1.857E-4 8.797E-7 7.7E-4 0.001096 0.001495 501 49500

10 2019

list(n=15253, Tpos=53)

list(Se=.99999999, Sp=0.996925, pi=0.0011032) #EDO 10 2019

**node mean sd MC error 2.5% median 97.5% start sample**

pi 0.001072 1.792E-4 8.13E-7 7.466E-4 0.001063 0.00145 501 49500

11 2019

list(n=15494, Tpos=51)

list(Se=.99999999, Sp=0.996925, pi=0.0011032) #EDO 11 2019

**node mean sd MC error 2.5% median 97.5% start sample**

pi 0.001052 1.749E-4 8.61E-7 7.35E-4 0.001042 0.001421 501 49500

12 2019

**node mean sd MC error 2.5% median 97.5% start sample**

pi 0.001042 1.745E-4 8.299E-7 7.263E-4 0.001034 0.001409 501 49500
